# Prevalence and Vascular Distribution of Multiterritorial Atherosclerosis Among Community-Dwelling Adults in Southeast China

**DOI:** 10.1001/jamanetworkopen.2022.18307

**Published:** 2022-06-27

**Authors:** Yuesong Pan, Jing Jing, Xueli Cai, Zening Jin, Suying Wang, Yilong Wang, Chunlai Zeng, Xia Meng, Jiansong Ji, Long Li, Lingchun Lyu, Zhe Zhang, Lerong Mei, Hao Li, Shan Li, Tiemin Wei, Yongjun Wang

**Affiliations:** 1 Department of Neurology, Beijing Tiantan Hospital, Capital Medical University, Beijing, China; 2 China National Clinical Research Center for Neurological Diseases, Beijing, China; 3 Department of Neurology, Lishui Hospital, Zhejiang University School of Medicine, Lishui, China; 4 Department of Cardiology, Beijing Tiantan Hospital, Capital Medical University, Beijing, China; 5 Cerebrovascular Research Lab, Lishui Hospital, Zhejiang University School of Medicine, Lishui, China; 6 Department of Cardiology, Lishui Hospital, Zhejiang University School of Medicine, Lishui, China; 7 Key Laboratory of Imaging Diagnosis and Minimally Invasive Intervention Research, Lishui Hospital, Zhejiang University School of Medicine, Lishui, China; 8 Heart Center and Beijing Key Laboratory of Hypertension, Beijing Chaoyang Hospital, Capital Medical University, Beijing, China; 9 Advanced Innovation Center for Human Brain Protection, Capital Medical University, Beijing, China; 10 Research Unit of Artificial Intelligence in Cerebrovascular Disease, Chinese Academy of Medical Sciences, Beijing, China; 11 Center for Excellence in Brain Science and Intelligence Technology, Chinese Academy of Sciences, Shanghai, China

## Abstract

**Importance:**

Data are limited on the prevalence and vascular distribution of multiterritorial atherosclerotic plaque and stenosis in community populations.

**Objective:**

To investigate the prevalence and vascular distribution of multiterritorial atherosclerotic plaque and stenosis in older, community-dwelling populations in China.

**Design, Setting, and Participants:**

This cross-sectional study was based on the baseline survey from the Polyvascular Evaluation for Cognitive Impairment and Vascular Events (PRECISE) study, a population-based prospective cohort study that enrolled community-dwelling adults aged 50 to 75 years based on cluster sampling from 6 villages and 4 living communities of Lishui city in southeast China. Data were collected from May 2017 to September 2019 and analyzed from September to November 2021.

**Main Outcomes and Measures:**

Atherosclerotic plaque and stenosis at baseline were assessed in multiple vascular territories. Brain vessel wall magnetic resonance imaging (MRI) for intracranial and extracranial arteries; computed tomography angiography (CTA) for coronary, subclavian, aorta, renal, and iliofemoral arteries; and ankle-brachial index for peripheral arteries were performed at baseline survey. The extent of atherosclerosis was assessed according to the number of these 8 vascular sites affected, and polyvascular lesions were defined as at least 2 affected sites.

**Results:**

A total of 3433 of 4202 invited individuals consented to participate in the study. After excluding 366 participants with contraindications for MRI or CTA scanning, with life expectancies of 4 years of fewer, or with mental disease, a total of 3067 community-dwelling adults were enrolled. The mean (SD) age was 61.2 (6.7) years; 1640 (53.5%) were women, and 74 (2.4%) had prevalent ASCVD. Most participants (2870 [93.6%]) had atherosclerotic plaques in at least 1 vascular territory. Atherosclerotic plaques were mostly detected in the aorta (2419 [79.6%]) and iliofemoral arteries (2312 [75.8%]), followed by subclavian (1500 [49.8%]), coronary (1366 [44.9%]), extracranial (1110 [36.4%]), renal (873 [28.7%]), and intracranial (542 [17.7%]) arteries. A substantial proportion of participants (1180 [38.5%]) had arterial stenosis of 50% or greater, predominantly affecting the coronary (542 [17.8%]) and iliofemoral (527 [17.3%]) arteries. Polyvascular atherosclerotic plaque was observed in 2541 participants (82.8%), with 1436 (46.8%) with plaque affecting 4 or more vascular territories, and polyvascular stenosis was observed in 412 patients (13.4%).

**Conclusions and Relevance:**

In this study, atherosclerotic plaque was highly prevalent in the older community population in China, and a substantial proportion of individuals reach stenosis of 50% or greater.

## Key Points

**Question:**

What are the prevalence, vascular distribution, and burden of atherosclerotic plaque and stenosis across multiple vascular territories in community-dwelling populations in China?

**Findings:**

In this cross-sectional study of 3067 adults in southeastern China, atherosclerotic plaque and stenosis were highly prevalent, with many individuals having atherosclerosis in multiple vascular territories.

**Meaning:**

These findings suggest that atherosclerosis screening and intensification of primary cardiovascular prevention might be needed for older adults in China.

## Teaser

This cross-sectional study investigates the prevalence and vascular distribution of multiterritorial atherosclerotic plaque and stenosis in community-dwelling older adults in China.

## Introduction

Atherosclerosis is the major underlying cause of cardiovascular disease, which is a leading cause of mortality worldwide.^1^ Polyvascular atherosclerosis with coexistent multiterritorial lesions was associated with a higher risk of future cardiovascular diseases.^2,3^ Previous studies have found that subclinical carotid atherosclerosis and coronary artery calcification increased the risk of vascular events.^4,5^ Approximately 7% of individuals older than 65 years were reported to have atherosclerotic renal artery stenosis, which may cause resistant hypertension and ischemic nephropathy.^6,7^ Other studies have shown that approximately one-third of the older community population has intracranial atherosclerosis.^8^ Compared with White individuals, the Asian population might have a higher prevalence of intracranial atherosclerosis, which contributes to more cases of ischemic stroke in the Asian population.^9,10^ Therefore, comprehensive evaluation of multiterritorial lesions is required to fully understand the distribution and burden of atherosclerosis and to precisely assess the risk of future cardiovascular and cerebrovascular events. However, previous studies either focused on individual arterial territories,^8,11,12,13^ on roughly defined polyvascular diseases according to established clinical diseases in multiple vascular territories,^2^ or on multiterritorial subclinical atherosclerosis without inclusion of intracranial arteries.^14^ There are limited data on the prevalence, vascular distribution, and burden of polyvascular atherosclerosis in the arterial system with intracranial, coronary, and peripheral vascular territories.

The Polyvascular Evaluation for Cognitive Impairment and Vascular Events (PRECISE) study is a population-based prospective cohort study with a comprehensive evaluation of atherosclerosis in multiple vascular territories using advanced vascular imaging techniques. In this cross-sectional study, we evaluated the prevalence, vascular distribution, and burden of polyvascular atherosclerosis in a community population based on the baseline survey of the PRECISE study.

## Methods

### Study Design and Participants

The rationale and design of the PRECISE study (NCT03178448) have been described.^15^ In brief, PRECISE is an ongoing population-based prospective cohort study to establish the prevalence of clinical or subclinical polyvascular lesions, the progression rate of plaque, and the association between polyvascular lesions and future events in community-dwelling older adults in China. Community-dwelling adults aged 50 to 75 years based on cluster sampling from 6 villages and 4 living communities of Lishui in southeastern China were consecutively recruited between May 2017 and September 2019. These 10 clusters were selected from 379 villages and 26 living communities in Liandu district, Lishui, based on convenience sampling. All eligible individuals in these selected villages and communities were invited and were excluded only when they could not be accessed during 3 attempts made on 3 days. A total of 3433 of 4202 invited individuals consented to participate in the study. After excluding 366 participants with contraindications for magnetic resonance imaging (MRI) or computed tomography angiography (CTA) scanning, with life expectancies of 4 years or fewer, or with mental disease, a total of 3067 individuals were enrolled in this study. Responders in the study had similar sex and age distribution as nonresponders (eTable 1 in the Supplement). Participants in the PRECISE study had similar demographic characteristics and major medical histories as nationwide data in China (eTable 2 in the Supplement). Participants were examined at baseline by physical examination, brain MRI, vascular MRI, thoracoabdominal CTA, and ankle-brachial index (ABI) and will be followed up at 2 and 4 years. The protocol was approved by the ethics committee at Beijing Tiantan Hospital and Lishui Hospital. All participants provided written informed consent before enrollment. This study followed the Strengthening the Reporting of Observational Studies in Epidemiology (STROBE) reporting guideline.

### Demographic Characteristics and Vascular Risk Factors Collection

Face-to-face clinical interviews and examinations were all performed at Lishui Hospital by trained research coordinators at baseline.^15^ Baseline demographic characteristics, medical history, family history, lifestyle, smoking and drinking status, and medication use were collected at the baseline survey.

Cardiovascular risk factors were determined from interviews and examinations as follows. First, smoking included former and current smoking status. Current smokers were defined as those who smoked at least 1 cigarette per day on average during the last month.^16^ Former smokers were defined as those who had ever smoked regularly and had stopped for more than 1 month. Second, a family history of cardiovascular disease was defined as at least a parent or a sibling with coronary heart disease or stroke.^17^ Hypertension was defined as systolic blood pressure of 140 mm Hg or greater, diastolic blood pressure of 90 mm Hg or greater, self-reported hypertension previously diagnosed by a physician, or current use of antihypertensive agents.^18,19^ Diabetes was defined as fasting plasma glucose of 126.1 mg/dL or greater (to convert to millimoles per liter, multiply by 0.0555), 2-hour postload glucose of 200 mg/dL or greater, hemoglobin A_1c_ of 6.5% or greater (to convert to proportion of total hemoglobin, multiply by 0.01), self-reported diabetes previously diagnosed by a physician, or current use of antidiabetic agents.^20,21^ Dyslipidemia was defined as total cholesterol 240 mg/dL or greater, low-density lipoprotein cholesterol of 160 mg/dL or greater, high-density lipoprotein cholesterol of less than 40 mg/dL (to convert cholesterol to millimoles per liter, multiply by 0.0259), or self-reported dyslipidemia previously diagnosed by a physician.^22,23^ Presence of atherosclerotic cardiovascular disease (ASCVD) was defined as history of ischemic stroke or myocardial infarction.

### Intracranial and Extracranial Vascular MRI

Intracranial and extracranial arteries were evaluated by MRI performed at baseline using a 3.0T scanner (Ingenia 3.0T [Philips]) by trained investigators based on a standardized protocol (eMethods 1 in the Supplement). MRI sequences included 3-dimensional time-of-flight magnetic resonance angiography (3-D–TOF MRA), 3-D isotropic high-resolution black-blood T1w vessel wall imaging, and simultaneous noncontrast angiography and intraplaque hemorrhage imaging.

MRI data were collected in DICOM format on discs and then analyzed by 2 raters who were masked to the participants’ information. Inconsistencies were settled by another senior neurologist (J.J.). The presence of atherosclerotic plaque was defined as eccentric wall thickening with or without luminal stenosis as seen on 3-D–TOF MRA or black-blood MR images.^8^ Lumen stenosis was assessed at the site of wall thickening identified on black-blood MR images (eMethods 2 in the Supplement). The presence of intracranial and carotid artery stenosis was defined as 50% to 99% stenosis or occlusion in the intracranial and carotid artery according to the Warfarin-Aspirin Symptomatic Intracranial Disease Trial criteria^24^ and the North American Symptomatic Carotid Endarterectomy Trial criteria,^25^ respectively.

### Thoracoabdominal CTA

Thoracoabdominal CTA for coronary, subclavian, aorta, renal, and iliofemoral arteries was performed at baseline using 1 dual-source CT scanner (SOMATOM Force [Siemens]) by trained investigators, based on a standardized protocol (eMethods 3 in the Supplement). Contrast medium iodixanol (320 mg I/mL [Visipaque, GE Healthcare]) was administered to perform CTA examination.

CTA data were collected in DICOM format on discs and then reconstructed and analyzed by 2 raters who were masked to the participants’ information at a cardiac image–viewing workstation in the Core Imaging Laboratory of Keya Medical Technology (Shenzhen, China). A multitask deep learning network (eMethods 4 in the Supplement) was used to automatically reconstruct the 3-D anatomical geometry of the input CTA images, and quantitative results for plaque and stenosis were calculated and characterized. An experienced imaging analyst then determined atherosclerosis based on the 3-D geometry, quantitative results, and CTA image (eMethods 4 in the Supplement). The presence of atherosclerotic plaque was defined as structures of at least a 1-mm^2^ area within or adjacent to the artery lumen and clearly distinguishable from the vessel lumen. For each territory, the area-based degree of narrowing was recorded for the most stenotic plaque. The presence of artery stenosis was defined as 50% to 99% stenosis or occlusion at thoracoabdominal arteries according to the Society of Cardiovascular Computed Tomography criteria.^26^

### ABI

The ABI was assessed to evaluate atherosclerotic peripheral arteries using Doppler ultrasound (Huntleigh Health Care Ltd) after a 10-minute rest in the supine position. ABI values of 0.9 or less were considered abnormal and with atherosclerosis in peripheral arteries.^27^

### Extent of Multiterritorial Atherosclerosis

Atherosclerosis was defined as the presence of at least 1 atherosclerotic plaque in the intracranial, extracranial, coronary, subclavian, aorta, renal, iliofemoral, or peripheral (ABI ≤0.9) arteries. The intracranial arteries assessed included the distal internal carotid, middle cerebral (M1 and M2), anterior cerebral (A1 and A2), posterior cerebral (P1 and P2), basilar, and vertebral (V4) arteries. Extracranial arteries assessed included the common carotid, proximal internal carotid, and vertebral (V1, V2, V3) arteries. Coronary arteries included the left main, left descending, left circumflex, obtuse margin, diagonal, septal branch, right coronary, posterior lateral branches, and right posterior descending arteries. Subclavian arteries included the left and right. Aorta arteries included arcus aortae and abdominal aorta. Renal arteries included the left and right. Iliofemoral arteries included the common iliac, internal iliac, and external iliofemoral arteries. The extent of atherosclerotic plaque and stenosis were assessed according to the number of these 8 vascular sites (ie, intracranial, extracranial, coronary, subclavian, aorta, renal, iliofemoral, and peripheral arteries) affected. Atherosclerosis in the symmetrical right and left arteries was counted as 1 if both sides were affected in territories such as extracranial, subclavian, renal, and ileofemoral arteries. The extent of atherosclerotic plaque and stenosis were classified as 0, 1, 2 to 3, or 4 to 8 vascular sites affected.^14^ Polyvascular arteriosclerotic lesions were defined as at least 2 affected sites (multiterritorial) in these arteries.^14^

### Statistical Analysis

Continuous variables are presented as the mean with SD or median with IQR, as appropriate, and categorical variables are presented as frequency with percentage. Baseline characteristics among sex and affected artery groups were compared by *t* test, Wilcoxon rank sum test, 1-way analysis of variance, or Kruskal-Wallis test for continuous variables as appropriate and χ^2^ test for categorical variables. Multivariable logistic regression models were used to examine the associations of age, sex, and cardiovascular risk factors with the presence of plaque or arterial stenosis in each vascular territory and the associations of the presence of atherosclerosis in individual vascular territories with the presence of ASCVD. Odds ratios with their 95% CIs were evaluated. Missing image data were not imputed, and analyses for each vascular territory were performed based on the available participants. A 2-sided *P* < .05 was considered statistically significant. All analyses were conducted with SAS version 9.4 (SAS Institute Inc). Data were analyzed from September 12 to November 30, 2021.

## Results

### Baseline Characteristics

A total of 3067 community-dwelling adults were enrolled in the PRECISE study at baseline between May 2017 and September 2019. There were 2 and 18 participants with noninterpretable MRI images in intracranial and extracranial arteries, respectively. Thoracoabdominal CTA was not performed in 15 participants because of CTA contraindication. Additionally, there were 7, 41, 14, 7, and 2 participants with noninterpretable CTA images in coronary, subclavian, aorta, renal, and iliofemoral arteries, respectively, and 20 participants without examination of ABI; 102 participants had missing data in at least 1 vascular territory. All participants had interpretable images in at least 1 vascular territory; thus, all the participants were included in this analysis.

Baseline characteristics and risk factors are presented in Table 1 and eTable 3 in the Supplement. The mean (SD) age of the participants was 61.2 (6.7) years; 1640 (53.5%) were women. The most prevalent traditional risk factor was hypertension (1321 [43.1%]), followed by dyslipidemia (1282 [41.8%]). Most participants (2546 [84.0%]) had at least 1 traditional risk factor, 892 (29.1%) had 2, and 654 (21.3%) had 3 or more risk factors. The prevalence of traditional risk factors was similar between men and women and increased with age, except smoking. Smoking was very prevalent in men (396 [27.8%] former smokers and 628 [44.0%] current smokers), but only 1 (0.1%) former and 1 (0.1%) current smokers were women. There were 74 participants (2.4%) with prevalent ASCVD.

**Table 1.** Demographic Characteristics and Cardiovascular Risk Factors

Characteristic	Participants, No (%)			*P* value
	Total (N = 3067)	Men (n = 1427)	Women (n = 1640)	
Age, mean (SD), y	61.2 (6.7)	61.5 (6.7)	61.0 (6.7)	.04
BMI				
Mean (SD)	23.8 (3.0)	23.8 (2.9)	23.8 (3.1)	.73
Overweight, 24.0-27.9	1113 (36.3)	522 (36.6)	591 (36.0)	.69
Obesity, ≥28.0	268 (8.7)	118 (8.3)	150 (9.1)	
Blood pressure, mean (SD), mm Hg				
Systolic	129.3 (16.3)	128.8 (15.6)	129.8 (17.0)	.11
Diastolic	75.2 (9.0)	76.7 (9.1)	73.9 (8.8)	<.001
Vascular risk factors				
Smoking	1026 (33.5)	1024 (71.8)	2 (0.1)	<.001
Former smoker	397 (12.9)	396 (27.8)	1 (0.1)	<.001
Current smoker	629 (20.5)	628 (44.0)	1 (0.1)	
Family history of ASCVD	616 (20.1)	280 (19.6)	336 (20.5)	.55
Hypertension	1321 (43.1)	596 (41.8)	725 (44.2)	.17
Diabetes	663 (21.6)	296 (20.7)	367 (22.4)	.27
Dyslipidemia	1282 (41.8)	598 (41.9)	684 (41.7)	.91
Treatment				
Antihypertensive therapy	823 (26.8)	343 (24.0)	480 (29.3)	.001
Antidiabetic therapy	274 (8.9)	111 (7.8)	163 (9.9)	.04
Lipid-lowering therapy	120 (3.9)	54 (3.8)	66 (4.0)	.73
ASCVD^a^	74 (2.4)	43 (3.0)	31 (1.9)	.04

Abbreviations: ASCVD, atherosclerotic cardiovascular disease; BMI, body mass index (calculated as weight in kilograms divided by height in meters squared).

^a^ ASCVD included history of ischemic stroke and myocardial infarction.

### Prevalence, Vascular Distribution, and Extent of Atherosclerotic Plaque and Arterial Stenosis

**Figure 1.**
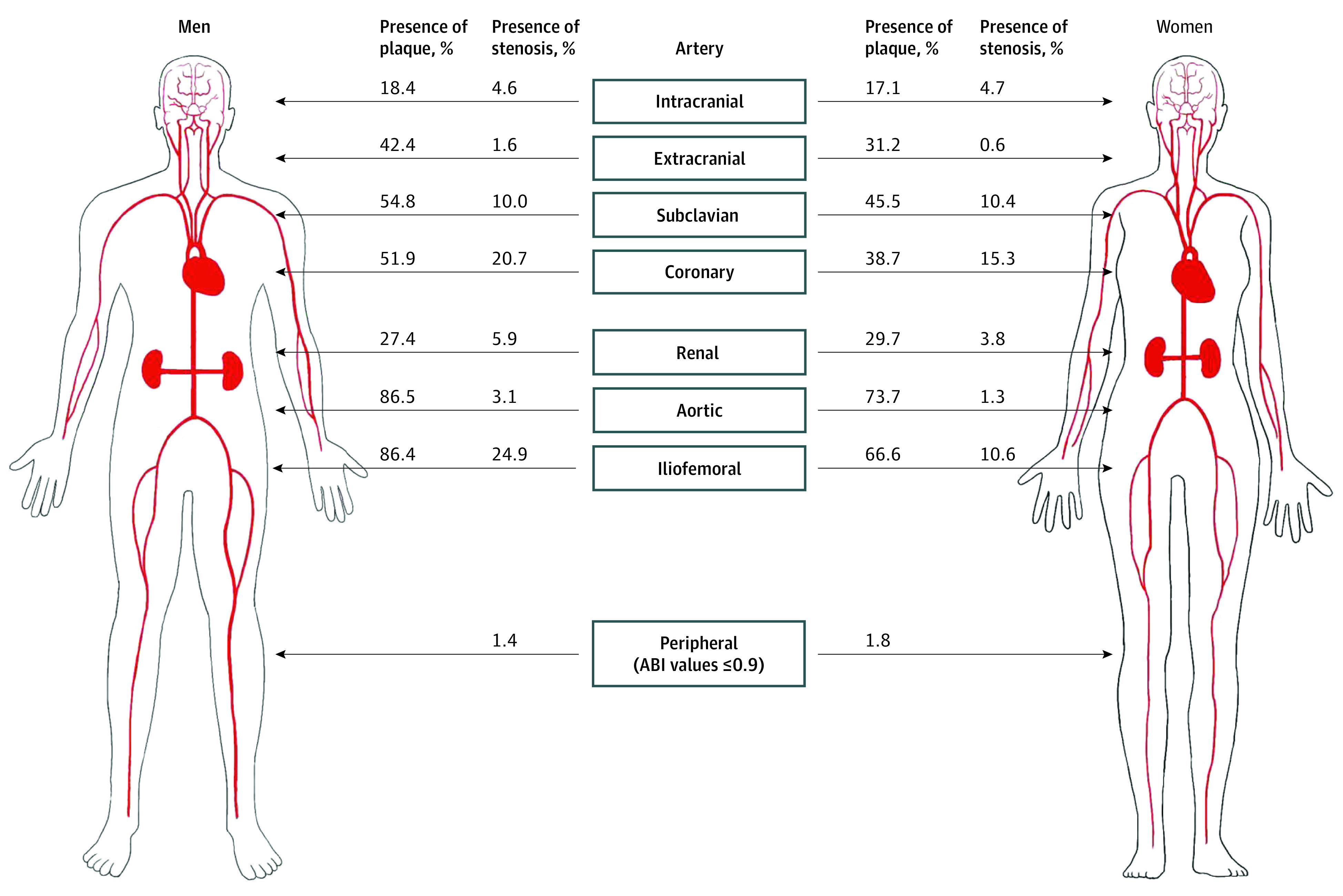
Prevalence and Vascular Distribution of Plaque and Arterial Stenosis ABI indicates ankle-brachial index.

Most participants (2870 [93.6%]) had atherosclerotic plaques in at least 1 vascular territory. Plaques were mostly detected in the aorta (2419 [79.6%]) and iliofemoral arteries (2312 [75.8%]), followed by the subclavian (1500 [49.8%]), coronary (1366 [44.9%]), extracranial (1110 [36.4%]; carotids, 1081 [35.5%], extracranial vertebral artery, 53 [1.7%]), renal (873 [28.7%]), and intracranial arteries (542 [17.7%]) (Figure 1). A substantial proportion of participants had arterial stenosis in at least 1 vascular territory (1180 [38.5%]), mostly affecting the coronary (542 [17.8%]) and iliofemoral (527 [17.3%]) arteries (Figure 1). Only 49 cases (1.6%) of abnormal ABI were detected. The prevalence of plaque and stenosis in individuals without a history of ASCVD (representing subclinical atherosclerosis) were 93.4% (2796 participants) and 37.7% (1127 participants), respectively, and similar vascular distributions were observed (eFigure 1 in the Supplement). A total of 453 participants (14.9%) and 752 participants (24.7%) had only noncalcified plaques and any plaque noncalcified in the coronary artery, respectively (eTable 4 in the Supplement). Severe forms of coronary atherosclerosis with significant stenosis in the left main, proximal left anterior descending, or 3-vessel disease were found in 203 participants (6.7%). Atherosclerosis was similarly prevalent in renal and intracranial arteries in men and in women but more prevalent in men than in women across all other vascular territories. The prevalence of atherosclerotic plaque and stenosis increased with age for both sexes and across all vascular territories (Table 2). Participants with atherosclerosis in each individual vascular territory had a higher prevalence of traditional risk factors except family history (eTables 5 and 6 in the Supplement).

**Table 2.** Prevalence of Plaque and Arterial Stenosis by Age and Sex in Each Vascular Territory

Territory	Total No.	Participants, No./total No. (%)									
		Plaque					Arterial stenosis				
		50-54 y	55-59 y	60-64 y	65-69 y	70-75 y	50-54 y	55-59 y	60-64 y	65-69 y	70-75 y
Intracranial artery											
Men	1426	38/304 (12.5)	55/325 (16.9)	58/337 (17.2)	70/284 (24.6)	41/176 (23.3)	9/304 (3.0)	9/325 (2.8)	16/337 (4.7)	22/284 (7.7)	10/176 (5.7)
Women	1639	37/374 (9.9)	48/419 (11.5)	64/369 (17.3)	67/287 (23.3)	64/190 (33.7)	7/374 (1.9)	12/419 (2.9)	11/369 (3.0)	24/287 (8.4)	23/190 (12.1)
Extracranial artery											
Men	1419	104/303 (34.3)	130/323 (40.2)	142/336 (42.3)	139/283 (49.1)	86/174 (49.4)	4/303 (1.3)	1/323 (0.3)	8/336 (2.4)	6/283 (2.1)	4/174 (2.3)
Women	1630	108/373 (29.0)	128/416 (30.8)	104/366 (28.4)	92/285 (32.3)	77/190 (40.5)	2/373 (0.5)	0	1/366 (0.3)	2/285 (0.7)	5/190 (2.6)
Coronary artery											
Men	1414	114/300 (38.0)	147/322 (45.7)	172/336 (51.2)	181/282 (64.2)	120/174 (69.0)	43/300 (14.3)	41/322 (12.7)	65/336 (19.3)	75/282 (26.6)	68/174 (39.1)
Women	1631	76/373 (20.4)	110/417 (26.4)	170/367 (46.3)	154/285 (54.0)	122/189 (64.6)	28/373 (7.5)	40/417 (9.6)	69/367 (18.8)	49/285 (17.2)	64/189 (33.9)
Subclavian artery											
Men	1396	105/295 (35.6)	157/322 (48.8)	184/329 (55.9)	190/279 (68.1)	129/171 (75.4)	15/295 (5.1)	24/322 (7.5)	30/329 (9.1)	42/279 (15.1)	29/171 (17.0)
Women	1615	84/371 (22.6)	146/413 (35.4)	196/363 (54.0)	170/281 (60.5)	139/187 (74.3)	15/371 (4.0)	29/413 (7.0)	44/363 (12.1)	35/281 (12.5)	45/187 (24.1)
Aortic artery											
Men	1412	234/300 (78.0)	267/323 (82.7)	290/334 (86.8)	265/281 (94.3)	165/174 (94.8)	4/300 (1.4)	9/323 (2.8)	12/334 (3.6)	12/281 (4.3)	7/174 (4.1)
Women	1626	202/373 (54.2)	282/416 (67.8)	281/364 (77.2)	252/285 (88.4)	181/188 (96.3)	0	2/416 (0.5)	7/364 (1.9)	5/285 (1.8)	7/188 (3.7)
Renal artery											
Men	1414	30/301 (10.0)	62/323 (19.2)	86/335 (25.7)	116/281 (41.3)	94/174 (54.0)	3/301 (1.0)	9/323 (2.8)	20/335 (6.0)	29/281 (10.3)	22/174 (12.6)
Women	1631	31/374 (8.3)	78/418 (18.7)	133/366 (36.3)	130/285 (45.6)	113/188 (60.1)	2/374 (0.5)	6/418 (1.4)	17/366 (4.6)	16/285 (5.6)	21/188 (11.2)
Iliofemoral artery											
Men	1416	227/301 (75.4)	273/323 (84.5)	301/336 (89.6)	257/282 (91.1)	165/174 (94.8)	40/301 (13.3)	73/323 (22.6)	80/336 (23.8)	91/282 (32.3)	69/174 (39.7)
Women	1634	160/374 (42.8)	244/418 (58.4)	271/368 (73.6)	242/285 (84.9)	172/189 (91.0)	4/374 (1.1)	22/418 (5.3)	41/368 (11.1)	46/285 (16.1)	61/189 (32.3)
Ankle-brachial index ≤0.9											
Men	1421	NA	NA	NA	NA	NA	7/302 (2.3)	3/324 (0.9)	5/336 (1.5)	4/284 (1.4)	1/175 (0.6)
Women	1626	NA	NA	NA	NA	NA	10/373 (2.7)	7/416 (1.7)	7/366 (1.9)	3/282 (1.1)	2/189 (1.1)

Abbreviation: NA, not applicable.

The presence of atherosclerosis in the aortic artery was more strongly associated with that in the renal, iliofemoral, and subclavian arteries than that in other arteries, whereas atherosclerosis in the iliofemoral artery was strongly associated with that in the aortic and renal arteries (Table 3). Having plaque in the aortic or iliofemoral arteries corresponded to an 85.3% probability of finding plaque in any other territory. There was moderate coexisting atherosclerosis among intracranial, renal, and coronary arteries. Among participants with plaques in the intracranial and renal arteries, 41.5% and 36.8% did not have coexisting plaques present in the coronary artery, respectively. The presence of atherosclerosis in individual vascular territories, except abnormal ABI, was associated with the presence of ASCVD (eTable 7 in the Supplement).

**Table 3.** Associations Between the Presence of Atherosclerosis in Individual Vascular Territories

Measure	Intracranial artery	Extracranial artery	Coronary artery	Subclavian artery	Aortic artery	Renal artery	Ilio-femoral artery	Ankle-brachial index ≤0.9
**Intracranial artery**								
PPV, %	NA	49.3	58.5	66.3	91.2	44.4	86.6	2.0
NPV, %	NA	66.3	58.0	53.7	22.8	74.7	26.5	98.5
OR (95% CI)^a^	NA	1.81 (1.50-2.20)	1.61 (1.32-1.97)	1.86 (1.51-2.29)	2.42 (1.75-3.35)	1.89 (1.54-2.33)	1.79 (1.35-2.36)	1.56 (0.79-3.12)
*P* value	NA	<.001	<.001	<.001	<.001	<.001	<.001	.20
**Extracranial artery**								
PPV, %	23.8	NA	50.6	59.8	85.3	38.0	82.0	2.0
NPV, %	86.0	NA	58.4	55.9	23.5	76.7	27.8	98.6
OR (95% CI)^a^	1.81 (1.50-2.20)	NA	1.26 (1.08-1.47)	1.74 (1.48-2.04)	1.54 (1.26-1.90)	1.99 (1.67-2.37)	1.49 (1.22-1.81)	1.55 (0.87-2.75)
*P* value	<.001	NA	.004	<.001	<.001	<.001	<.001	.13
**Coronary artery**								
PPV, %	22.8	40.9	NA	60.1	89.6	40.4	88.5	1.5
NPV, %	86.8	67.4	NA	58.6	28.5	80.8	34.6	98.3
OR (95% CI)^a^	1.61 (1.32-1.97)	1.26 (1.08-1.47)	NA	1.55 (1.32-1.81)	2.37 (1.91-2.94)	2.16 (1.82-2.58)	2.78 (2.26-3.41)	1.03 (0.56-1.88)
*P* value	<.001	.004	NA	<.001	<.001	<.001	<.001	.93
**Subclavian artery**								
PPV, %	23.5	43.6	54.1	NA	92.5	43.3	88.1	1.3
NPV, %	88.1	70.9	64.4	NA	33.6	85.8	36.3	98.1
OR (95% CI)^a^	1.86 (1.51-2.29)	1.74 (1.48-2.04)	1.55 (1.32-1.81)	NA	4.56 (3.63-5.74)	3.52 (2.92-4.24)	2.91 (2.38-3.55)	0.86 (0.47-1.58)
*P* value	<.001	<.001	<.001	NA	<.001	<.001	<.001	.62
**Aortic artery**								
PPV, %	20.2	38.8	50.6	58.0	NA	34.5	84.8	1.7
NPV, %	92.4	73.8	77.0	81.9	NA	94.0	59.1	98.5
OR (95% CI)^a^	2.42 (1.75-3.35)	1.54 (1.26-1.90)	2.37 (1.91-2.94)	4.56 (3.63-5.74)	NA	6.06 (4.26-8.62)	5.53 (4.50-6.81)	1.48 (0.70-3.16)
*P* value	<.001	<.001	<.001	<.001	NA	<.001	<.001	.31
**Renal artery**								
PPV, %	27.3	48.1	63.2	75.2	95.8	NA	94.4	1.5
NPV, %	86.3	68.5	62.5	60.4	26.9	NA	31.7	98.3
OR (95% CI)^a^	1.89 (1.54-2.33)	1.99 (1.67-2.37)	2.16 (1.82-2.58)	3.52 (2.92-4.24)	6.06 (4.26-8.62)	NA	5.91 (4.31-8.10)	1.16 (0.58-2.29)
*P* value	<.001	<.001	<.001	<.001	<.001	NA	<.001	.68
**Iliofemoral artery**								
PPV, %	20.1	39.4	52.4	57.8	89.0	35.7	NA	1.6
NPV, %	90.2	73.1	78.7	75.4	49.9	93.4	NA	98.4
OR (95% CI)^a^	1.79 (1.35-2.36)	1.49 (1.22-1.81)	2.78 (2.26-3.41)	2.91 (2.38-3.55)	5.53 (4.50-6.81)	5.91 (4.31-8.10)	NA	1.33 (0.66-2.68)
*P* value	<.001	<.001	<.001	<.001	<.001	<.001	NA	.43
**Ankle-brachial index ≤0.9**								
PPV, %	22.4	44.9	40.8	41.7	81.6	26.5	75.5	NA
NPV, %	82.3	63.7	55.0	50.1	20.4	71.3	24.2	NA
OR (95% CI)^a^	1.56 (0.79-3.12)	1.55 (0.87-2.75)	1.03 (0.56-1.88)	0.86 (0.47-1.58)	1.48 (0.70-3.16)	1.16 (0.58-2.29)	1.33 (0.66-2.68)	NA
*P* value	.20	.13	.93	.62	.31	.68	.43	NA

Abbreviations: OR, odds ratio; NA, not applicable; NPV, negative predictive value; PPV, positive predictive value.

^a^ ORs were calculated using logistic regression models with presence of atherosclerosis in the artery in the column title (eg, extracranial artery) as the dependent variable and presence of atherosclerosis in the artery of the row title (eg, intracranial artery) as the independent variable, adjusted for age and sex.

**Figure 2.**
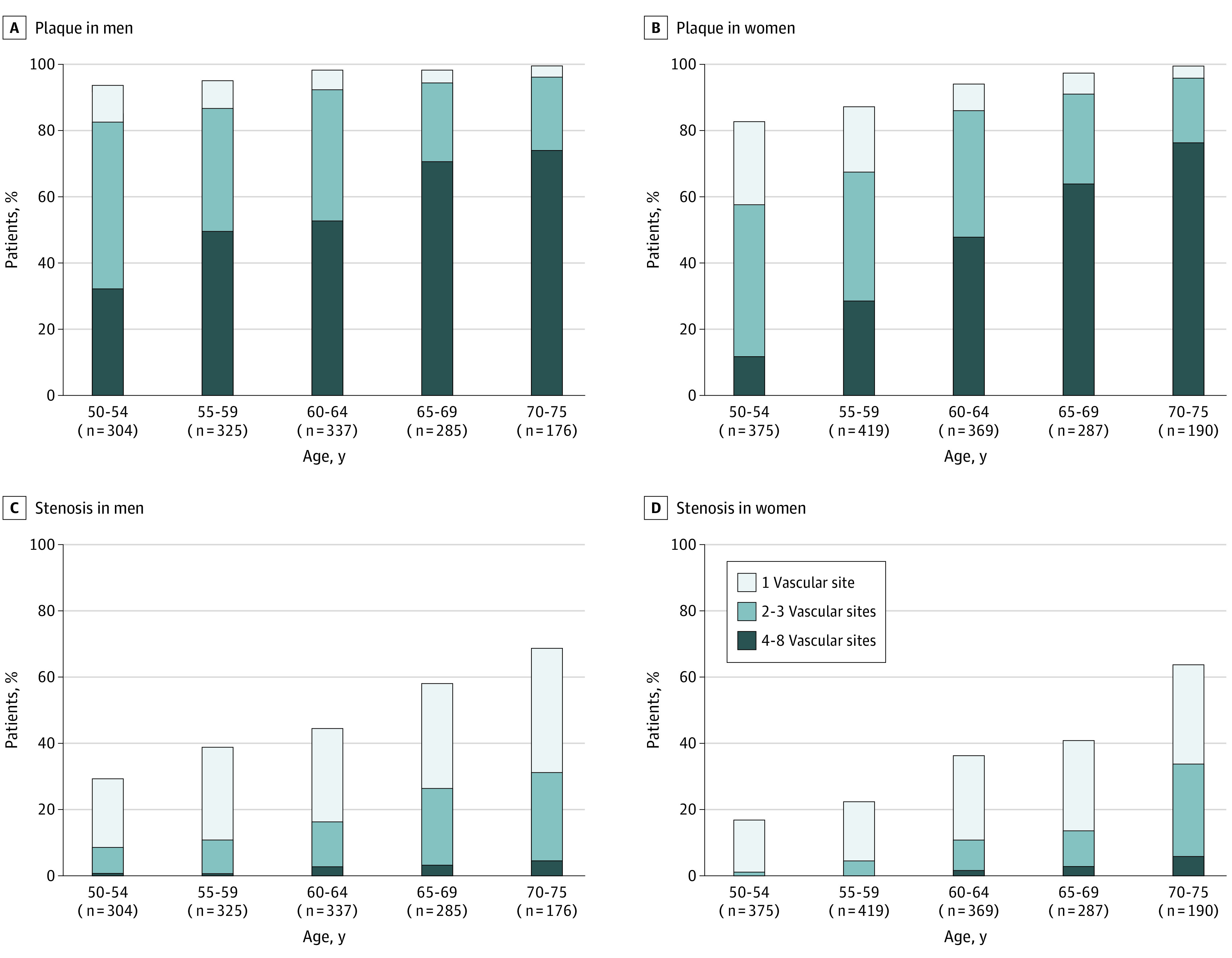
Burden of Multiterritorial Plaque and Arterial Stenosis by Age and Sex

Classification of participants according to the extent of atherosclerosis showed plaque with 1 vascular site affected in 10.7% (329 participants), plaque with 2 to 3 sites in 36.0% (1105 participants), and plaque with 4 to 8 sites in 46.8% (1436 participants), whereas the proportions of stenosis were 25.0% (768 participants), 11.6% (357 participants), and 1.8% (55 participants), respectively. The prevalence of polyvascular atherosclerosis was greater in men and increased with age, both in the entire population (Figure 2) and in those without a history of ASCVD (eFigure 2 in the Supplement). Notably, the extent of plaque in men was similar to that in women 5 to 10 years older. Those with polyvascular territories affected had a higher prevalence of all traditional risk factors except family history (eTables 8 and 9 in the Supplement).

## Discussion

In the population-based survey of the PRECISE study, more than 90% of community-dwelling older Chinese adults had atherosclerotic plaque in at least 1 vascular territory, with approximately 80% in polyvascular territories, and a substantial number of individuals (nearly 40%) reached arterial stenosis of at least 50%. The aorta and iliofemoral and coronary arteries were the most frequently affected vascular territories, and there was also a nonnegligible prevalence of atherosclerosis in the intracranial and renal arteries.

The prevalence and burden of atherosclerosis in the general population have been reported in several studies, and the estimate of polyvascular atherosclerosis ranges from 3% to 42%.^3,13,14,28^ The Swedish Cardiopulmonary Bioimage Study (SCAPIS)^13^ reported a higher prevalence of coronary CTA–detected atherosclerosis (48.5%) but a slightly lower prevalence of stenosis of at least 50% (6.5%) in adults aged 55 to 64 years than that in our study (41.5% and 14.9%, respectively). Most importantly, the Progression of Early Subclinical Atherosclerosis (PESA) study found that 63% of the middle-aged population had subclinical atherosclerosis, and 41% had multiterritorial atherosclerosis,^14^ which was much lower than that in our study (93.4% and 82.5%, respectively). Differences in participant age range and other characteristics, population sampling, examination techniques, and number of examined vascular territories may contribute to the heterogeneity of these estimates. PESA included middle-aged (ie, 40 to 54 years), predominantly male white-collar workers, whereas PRECISE recruited a relatively representative community-dwelling older (ie, 50 to 75 years) adult sample.^15^ Among the overlapping age range of 50 to 54 years, atherosclerosis in PESA vs PRECISE was higher in carotid arteries (48% in men and 35% in women vs 34% and 29%, respectively), similar in coronary (43% in men and 10% in women vs 38% and 20%, respectively), and iliofemoral (72% in men and 42% in women vs 75% and 43%, respectively) arteries but lower in aortic arteries (40% in men and 35% in women vs 78% and 54%, respectively). Furthermore, in PESA, detection of plaques by vascular ultrasonography may have been limited by the penetration of the vascular probe and the presence of air, and coronary artery calcium may leave out noncalcified plaques.^14^ In contrast, the advanced vascular MRI and CTA imaging techniques used in PRECISE may help to precisely evaluate vascular atherosclerosis. The PRECISE study found a large burden of atherosclerosis in the arterial system, and a substantial proportion reached stenosis of at least 50%. This indicates that atherosclerosis screening and intensification of primary cardiovascular prevention might be required for this population. As with a previous study,^14^ this study observed a similar association of increased atherosclerosis prevalence with male sex and age, and the risk of atherosclerosis in men was similar to that in women 5 to 10 years older. This may help to determine the time for atherosclerosis screening and intensification of primary cardiovascular prevention.

To our knowledge, few studies have investigated the prevalence and vascular distribution of atherosclerotic plaque and stenosis across multiple vascular territories in community populations.^3,4,14,29^ The Multi-Ethnic Study of Atherosclerosis (MESA) only evaluated coronary artery calcium and carotid intima-media thickness.^4^ The Atherosclerosis Risk in Communities (ARIC) study focused on carotid territories assessed by ultrasonography in the entire cohort and intracranial territories assessed by vessel wall MR imaging in a subgroup of 1980 participants.^8,29^ The PESA study evaluated subclinical atherosclerosis in the carotid, aortic, and iliofemoral territories using vascular ultrasonography and examined coronary artery calcium using CT in asymptomatic middle-aged individuals.^14^ The SCAPIS study assessed atherosclerosis in coronary arteries with CTA and in carotid arteries with ultrasonography and ABI.^13,30^ Importantly, the PRECISE study evaluated atherosclerosis across multiple vascular territories using advanced vascular imaging techniques, which might be relevant for a comprehensive overview of the distribution and burden of atherosclerosis in the arterial system. This may help to precisely evaluate atherosclerosis and may have the potential to help individualized risk assessments of future cerebrovascular events. In addition to the iliofemoral and aortic arteries,^14^ this study also found that there was moderate coexisting atherosclerosis among intracranial, renal, and coronary arteries. Intracranial atherosclerotic stenosis is a highly prevalent cause of stroke.^31^ The prevalence of intracranial atherosclerosis in our study was similar to that in another survey in China^32^ but slightly lower than that in the ARIC study, in which participants were older.^8^ Like a previous study,^6^ a nonnegligible proportion of individuals in our study had atherosclerosis and even stenosis at the renal artery, which may be associated with resistant hypertension and ischemic nephropathy. These vascular territories were not traditionally included in the routine physical examination but were of significance for comprehensive evaluation of atherosclerosis and primary cardiovascular disease prevention.

### Limitations

There were several limitations in this study. First, this study was a cross-sectional analysis of the baseline survey of the PRECISE cohort and therefore cannot investigate atherosclerosis progression and clinical events. Longitudinal follow-up data of the cohort may provide us with an opportunity to study the association of atherosclerosis with clinical events. Second, despite being representative in general, potential selection bias was unavoidable in this study because the population was selected from a single community region. Further investigation is required with a large sample size. Third, interpretation of the results should be made with caution and based on different examination methods. MRI and CTA imaging have differences in spatial resolution, which leads to differences in the sensitivity of detecting atherosclerosis and affects the comparison between different vascular territories. ABI of less than 0.9 was used to identify relatively severe atherosclerosis in peripheral arteries, which may not be comparable with imaging techniques. Fourth, lifelong dose of tobacco was not recorded in this study, which may cause misclassification of smoking.^14^ Fifth, the population in this cohort was restricted to older Chinese adults. Given the potential variation of disease profiles across ethnic groups, further investigation is required before extrapolation to other cohorts, especially in other racial and ethnic populations.

## Conclusions

With comprehensive evaluation of multiple vascular territories using advanced vascular imaging techniques, we found a high prevalence and burden of atherosclerosis in the arterial system, and a substantial proportion of participants reached stenosis of at least 50%. This study set the basis for the evaluation of atherosclerosis progression and risk stratification for future vascular events with longitudinal follow-up data.

## Notes

Supplement. **eMethods 1.** MRI Parameters **eMethods 2.** Evaluation of Plaque and Stenosis in Intracranial and Extracranial Arteries **eMethods 3.** Thoracoabdominal CTA Scan **eMethods 4. **Evaluation of Plaque and Stenosis in Coronary, Subclavian, Aorta, Renal and Iliofemoral Arteries **eFigure 1.** Prevalence and Vascular Distribution of Plaque and Arterial Stenosis in Individuals Without History of ASCVD **eFigure 2.** Burden of Multiterritorial Plaque and Arterial Stenosis by Age and Sex in Individuals Without History of ASCVD **eTable 1.** Comparison of Participant Characteristics of Responders and Nonresponders for the PRECISE Study **eTable 2.** Comparison of Participant Characteristics in the PRECISE Study and Nationwide Data **eTable 3.** Prevalence of Cardiovascular Risk Factors and ASCVD, Stratified by Age and Sex **eTable 4.** Distribution and Characteristics of Plaque and Stenosis in Thoracoabdominal Arteries **eTable 5.** Associations of Age, Sex, Cardiovascular Risk Factors With Presence of Plaque in Individual Vascular Territories **eTable 6.** Associations of Age, Sex, Cardiovascular Risk Factors With Presence of Arterial Stenosis in Individual Vascular Territories **eTable 7.** Associations Between the Presence of Atherosclerosis in Individual Vascular Territories and ASCVD **eTable 8.** Demographic Characteristics and Risk Factors by Number of Vascular Territories With Plaque **eTable 9.** Demographic Characteristics and Risk Factors by Number of Vascular Territories With Arterial Stenosis
eReferences.
