# Supplementary material for: Prevalence and Vascular Distribution of Multiterritorial Atherosclerosis Among Community-Dwelling Adults in Southeast China
Source: JAMA Netw Open. 2022 Jun 27;5(6):e2218307. doi: 10.1001/jamanetworkopen.2022.18307 (PMC9237794; doi:10.1001/jamanetworkopen.2022.18307)
Supplement: Supplement. — eMethods 1. MRI Parameters eMethods 2. Evaluation of Plaque and Stenosis in Intracranial and Extracranial Arteries eMethods 3. Thoracoabdominal CTA Scan eMethods 4. Evaluation of Plaque and Stenosis in Coronary, Subclavian, Aorta, Renal and Iliofemoral Arteries eFigure 1. Prevalence and Vascular Distribution of Plaque and Arterial Stenosis in Individuals Without History of ASCVD eFigure 2. Burden of Multiterritorial Plaque and Arterial Stenosis by Age and Sex in Individuals Without History of ASCVD eTable 1. Comparison of Participant Characteristics of Responders and Nonresponders for the PRECISE Study eTable 2. Comparison of Participant Characteristics in the PRECISE Study and Nationwide Data eTable 3. Prevalence of Cardiovascular Risk Factors and ASCVD, Stratified by Age and Sex eTable 4. Distribution and Characteristics of Plaque and Stenosis in Thoracoabdominal Arteries eTable 5. Associations of Age, Sex, Cardiovascular Risk Factors With Presence of Plaque in Individual Vascular Territories eTable 6. Associations of Age, Sex, Cardiovascular Risk Factors With Presence of Arterial Stenosis in Individual Vascular Territories eTable 7. Associations Between the Presence of Atherosclerosis in Individual Vascular Territories and ASCVD eTable 8. Demographic Characteristics and Risk Factors by Number of Vascular Territories With Plaque eTable 9. Demographic Characteristics and Risk Factors by Number of Vascular Territories With Arterial Stenosis eReferences. [file jamanetwopen-e2218307-s001.pdf]

Supplemental Online Content

Pan Y, Jing J, Cai X, et al. Prevalence and vascular distribution of multiterritorial atherosclerosis among community-dwelling adults in southeast China. *JAMA Netw Open.* 2022;5(6):e2218307. doi:10.1001/jamanetworkopen.2022.18307

eMethods 1. MRI Parameters

eMethods 2. Evaluation of Plaque and Stenosis in Intracranial and Extracranial Arteries

eMethods 3. Thoracoabdominal CTA Scan

eMethods 4. Evaluation of Plaque and Stenosis in Coronary, Subclavian, Aorta, Renal and Iliofemoral Arteries

eFigure 1. Prevalence and Vascular Distribution of Plaque and Arterial Stenosis in Individuals Without History of ASCVD

eFigure 2. Burden of Multiterritorial Plaque and Arterial Stenosis by Age and Sex in Individuals Without History of ASCVD

eTable 1. Comparison of Participant Characteristics of Responders and Nonresponders for the PRECISE Study

eTable 2. Comparison of Participant Characteristics in the PRECISE Study and Nationwide Data

eTable 3. Prevalence of Cardiovascular Risk Factors and ASCVD, Stratified by Age and Sex

eTable 4. Distribution and Characteristics of Plaque and Stenosis in Thoracoabdominal Arteries

eTable 5. Associations of Age, Sex, Cardiovascular Risk Factors With Presence of Plaque in Individual Vascular Territories

eTable 6. Associations of Age, Sex, Cardiovascular Risk Factors With Presence of Arterial Stenosis in Individual Vascular Territories

eTable 7. Associations Between the Presence of Atherosclerosis in Individual Vascular Territories and ASCVD

eTable 8. Demographic Characteristics and Risk Factors by Number of Vascular Territories With Plaque

eTable 9. Demographic Characteristics and Risk Factors by Number of Vascular Territories With Arterial Stenosis

eReferences.

This supplemental material has been provided by the authors to give readers additional information about their work.

© 2022 Pan Y et al. *JAMA Network Open.*

eMethods 1. MRI Parameters

Subjects were scanned on a 3.0T MRI scanner (Ingenia; Philips, Best, the Netherlands). The multi-contrast MRI protocol includes structural brain MRI, functional brain MRI and vascular MRI. Structural and functional brain MRI includes three dimensional T1-weighted magnetization-prepared rapid acquisition gradient-echo (T1w MPRAGE), two dimensional T2-weighted (T2w), two-dimensional fluid-attenuated inversion recovery (FLAIR), diffusion weighted imaging (DWI), susceptibility-weighted imaging (SWI), three-dimensional time-of-flight magnetic resonance angiography (TOF MRA), resting-state fMRI and diffusion tensor imaging (DTI). Vascular MRI for intracranial and carotidal arteries includes 3D isotropic high-resolution black-blood T1w vessel wall imaging (VWI) and Simultaneous Non-contrast Angiography and intraPlaque haemorrhage (SNAP) imaging. The detailed scanner parameters are listed as following:

Contrast	Orientation	Voxel /mm^3	Parameters	Scan time
TOF MRA	3D transverse	0.34×0.34×0.70	TE=3.5ms, TR=25ms, Flip angle=20°, 5 slabs	3:32
T1w MPRAGE	3D sagittal	1.00×1.00×1.00	TE=3.0ms, TR=6.7ms, TI=880ms, shot interval=2000ms, Flip angle=8°	4:30
fMRI	2D transverse	2.92×2.92×4.00	TE=30ms, TR=2100ms, Flip angle=80°, 180 scans with 8 dummy scans, SPIR fat suppression, resting state with eyes open	6:35
DTI	2D transverse	1.88×1.88×2.50	TE=90ms, TR=3500ms, b=0 and b=1000s/mm^2 with 32 diffusion encoding directions, SPIR fat suppression	4:05
intracranial T1w VWI	3D transverse	0.30×0.30×0.30	TE=20ms, TR=800ms, 3D TSE factor =30, Refocusing angle=50°, SPIR fat suppression	6:18
carotidal T1w VWI	3D coronal	0.40×0.40×0.40	TE=31ms, TR=600ms, 3D TSE factor =28, Refocusing angle=35°, SPIR fat suppression	3:03
SWI	3D axial	0.63×0.63×0.80	first TE=7.2ms, echo spacing = 6.2ms, 5 echoes, Flip angle=17°, TR =37ms	2:20
FLAIR imaging	2D axial	0.53×0.53×6.50	TE=110ms, TR=7000ms, TI=2300ms, SPIR fat suppression	2:27
T2w imaging	2D axial	0.51×0.51×6.50	TE=105ms, TR=2500ms, SPIR fat suppression	1:05
DWI	2D axial	1.20×1.20×6.50	TE=98ms, TR=2500ms, b=1000s/mm^2, ADC and eADC calculated online	0:30
intracranial SNAP	3D transverse	0.40×0.40×0.40	TE=6.4ms, TR=11ms, TI=510ms, shot interval=1025ms, Flip angle=11°/5°(PSIR), Fat suppression.	6:35
carotidal SNAP	3D coronal	0.28×0.28×0.40	TE=4.8ms, TR=10ms, TI=510ms, shot interval=1025ms, Flip angle=11°/5°(PSIR), Fat suppression.	5:17

© 2022 Pan Y et al. *JAMA Network Open.*

eMethods 2. Evaluation of Plaque and Stenosis in Intracranial and Extracranial Arteries

MRI data were collected in DICOM format on discs and then analyzed by two raters who were blinded to the participants' information (D.Y and H.L) at the Core Imaging Laboratory of the Imaging Research Center at Beijing Tiantan Hospital. Images of poor quality or technical failures were excluded from the analyzed samples. The presence of atherosclerotic plaque was defined as eccentric wall thickening with or without luminal stenosis as seen on 3D-TOF MRA or black blood MR images.[1] If no narrowing was identified on 3D-TOF MRA, the plaque was considered to have no detectable stenosis. The number of detectable plaques in each vascular territory was recorded.[1] Lumen stenosis was assessed at the site of wall thickening identified on black blood MR images. The presence of intracranial artery stenosis was defined as 50%-99% stenosis or occlusion of the intracranial artery according to the Warfarin-Aspirin Symptomatic Intracranial Disease Trial [WASID] criteria.[2] The presence of carotid artery stenosis was defined as 50%-99% stenosis or occlusion of the carotid artery according to the North American Symptomatic Carotid Endarterectomy Trial [NASCET] criteria.[3] Arterial stenosis was assessed at the site of the most severe degree of stenosis. The interobserver agreement for the detection of plaque and stenosis was studied by replicating the assessment of all participants by the two raters. Inconsistencies were determined by another senior neurologist (J.J). Good reproducibility was found for the presence of plaque and artery stenosis (Cohen κ= 0.97 and 0.79 for intracranial artery and Cohen κ= 0.94 and 0.86 for extracranial artery) in all territories.

© 2022 Pan Y et al. *JAMA Network Open.*

Intracranial artery evaluation:

Intracranial artery plaque and stenosis were evaluated by both 3D-TOF MRA and 3D isotropic high-resolution black-blood T1w vessel wall imaging (3D VISTA T1 weighted sequence, 0.6 mm × 0.6mm × 0.6 mm isotropic). We used Warfarin-Aspirin Symptomatic Intracranial Disease (WASID) method to evaluate the plaque and stenosis in intracranial artery.[2] The diameter of the artery was evaluated by vessel wall imaging. Percent stenosis = [(1- (diameter of stenosis / diameter of normal))] ×100%, where diameter of stenosis = the diameter of the artery at the site of the most severe degree of stenosis, and diameter of normal = the diameter of the proximal normal artery. If the proximal artery was diseased (e.g., middle cerebral artery origin stenosis), the diameter of the distal portion of the artery at its widest, parallel, non-tortuous normal segment was substituted (second choice).

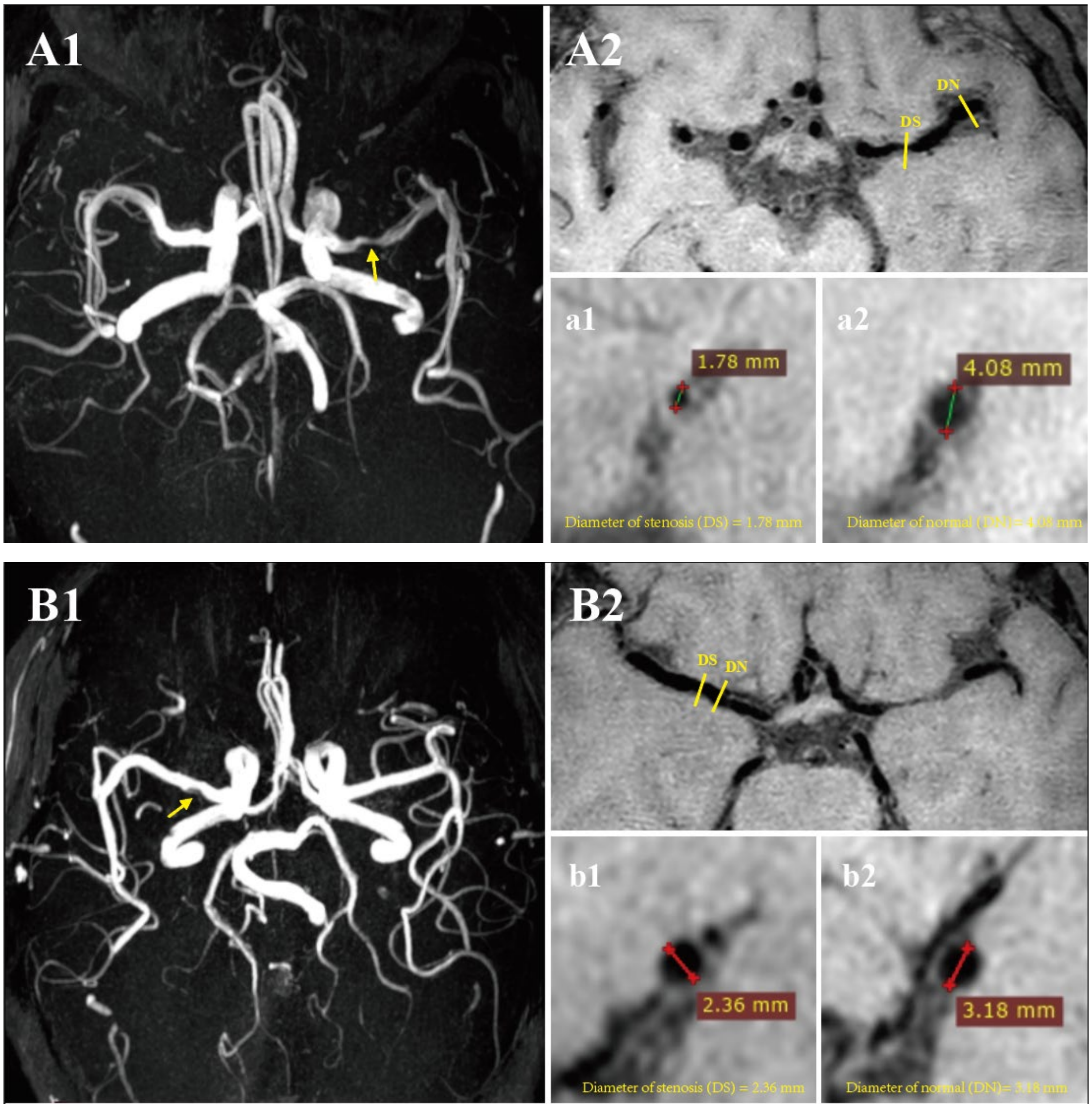

Figure A: TOF-MRA showed stenosis left middle artery of M1 segment. Percent stenosis = [(1- (1.78mm / 4.08mm))] ×100% = 56.4%;
Figure B: TOF-MRA showed stenosis right middle artery of M1 segment. Percent stenosis = [(1- (2.36mm / 3.18mm))] ×100% = 25.7%.

© 2022 Pan Y et al. *JAMA Network Open.*

Extracranial artery evaluation：

Extracranial artery plaque and stenosis were evaluated 3D isotropic high-resolution black-blood T1w vessel wall imaging (3D VISTA T1 weighted sequence, 0.8 mm × 0.8mm × 0.8 mm isotropic). We used the North American Symptomatic Carotid Endarterectomy Trial (NASCET) method to evaluate the plaque and stenosis in extracranial artery.[3] The diameter of the artery was evaluated by vessel wall imaging. Percent stenosis = [(1- (diameter of stenosis / diameter of normal))] ×100%, where diameter of stenosis = the diameter of the artery at the site of the most severe degree of stenosis, and diameter of normal = the diameter of the distal portion normal of the artery.

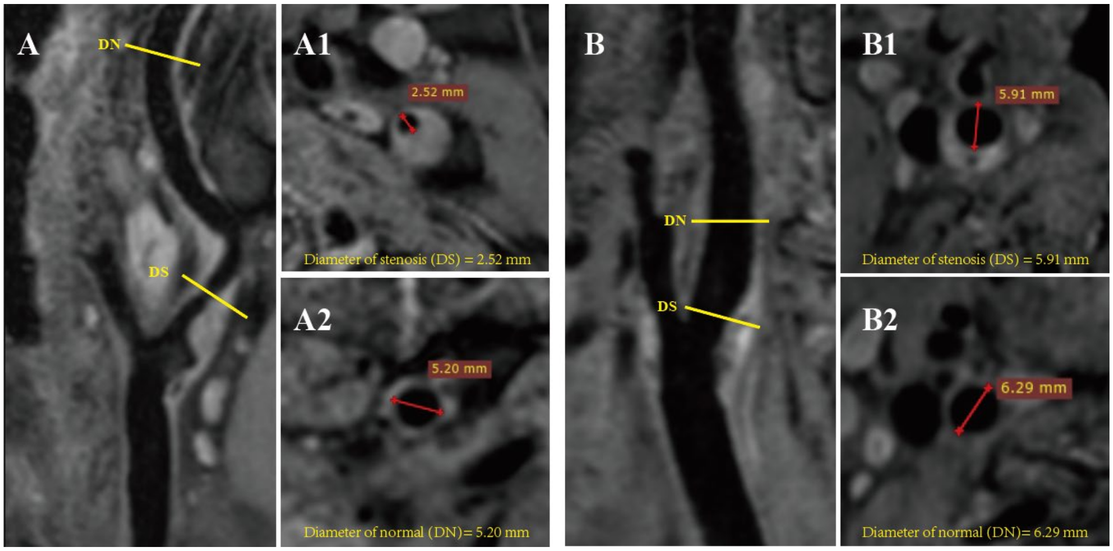

Figure A: Vessel wall imaging showed stenosis in C1 segment of carotid artery. Percent stenosis = [(1- (2.52mm /5.20mm))] ×100% =51.5%;
Figure B: Vessel wall imaging showed stenosis in C1 segment of carotid artery. Percent stenosis = [(1- (5.91mm / 6.29mm))] ×100% =6.0%.

© 2022 Pan Y et al. *JAMA Network Open.*

eMethods 3. Thoracoabdominal CTA Scan

Thoracoabdominal CTA for coronary, subclavian, aorta, renal and iliofemoral arteries was performed at baseline using one dual-source CT scanner (SOMATOM Force, Siemens, Germany) by trained investigators, based on a standardized protocol. In preparation for CTA imaging, renal function and potential contraindications were assessed to exclude participants for whom administration of contrast media could pose a risk. Each participant needed to rest before CTA examination to control the heart rate. Contrast medium iodoxanol (320 mg I/mL; Visipaque, GE Healthcare) was administered to perform CTA examination. A Siemens CARE Dose 4D automatic exposure control system was used to optimize the radiation dose in the examination process. The median total dose-length product (DLP) was 988 mGy-cm in this study.

© 2022 Pan Y et al. *JAMA Network Open.*

eMethods 4. Evaluation of Plaque and Stenosis in Coronary, Subclavian, Aorta, Renal and Iliofemoral Arteries

CTA data were collected in DICOM format on discs and then reconstructed and analyzed by two raters (Z.ZQ and Z.ZX) at a cardiac image-viewing workstation in the Core Imaging Laboratory of Keya Medical Technology (Shenzhen, China). Operators and readers were blinded to the participants' information.

Analysis of CTA image was conducted following 3 main steps: firstly, imaging processing software developed at Core Imaging Laboratory of Keya Medical Technology (Shenzhen, China) was used to automatically reconstruct 3D anatomical geometry of the input CTA imaging (aorta, coronary arteries, renal arteries, etc.). The software performs a set of imaging process algorithms that include both well-validated deep learning methods and traditional imaging processing technique. A multi-task deep learning network was used to extract the vessels from CTA images. This multi-task network simultaneously predicts the voxel-wise segmentation for the vessel and the distance maps of the segmentation. Meanwhile, the branch endpoints were also detected in order to generate the centerline. The segmentation mask was further refined based on the centerline generated from the distance map and the branch endpoints.[4, 5] Once the segmentation and the centerline were extracted, the cross section of vessel perpendicular to the centerline normal direction at each centerline point was extracted and the cross-sectional area was calculated based on the lumen mask. Next, experienced imaging analyst at the core lab reviewed the reconstructed geometry with the CTA image, and conducted geometry modification if necessary to ensure accurate 3D anatomical geometry. Finally, the atherosclerotic plaque and stenosis was analyzed by the two readers with both visual inspection from 3D anatomical geometry and CTA imaging, along with quantitative results provided by the software (such as stenosis degree). After CTA image processing by the software and experienced imaging analyst, quantitative results for plaque and stenosis can also be calculated and characterized by the software. With these information, reader could determine the final result based on the 3D geometry, quantitative results, and CTA image. Usually, CTA image has a transversal resolution of 0.4 mm, which would be the minimal diameter that the software could possibly handle. In the analysis procedure, the readers assessed the coronary arteries down to 1.5 mm of diameter according to the Standards of appropriate utilization and diagnostic reporting on coronary CT angiography- coronary artery disease-reporting and data system in China.

The software uses a set of trained algorithms specifically developed for each vascular territory. For instance, there is a trained and well-validated model for coronary arteries, while another model would be selected to process aorta. The model selection is done automatically as a pre-step by the software.

Images with poor quality or technical failures were excluded from the analyzed sample. Deep learning-based algorithms emerged as promising approaches for solving image analysis problems and have been successfully implemented for metal artifact reduction in CT imaging.[6] The software implements a deep-learning based algorithm to correct calcium blooming artifact. The algorithm uses DSA imaging as ground-truth, and trained the model to correlate CTA calcium reconstruction with ground-truth lumen from DSA. This correction was found to improve the accuracy of CTA lumen geometry reconstruction to be closer to the ground-truth DSA.

The presence of atherosclerotic plaque was defined as structures of at least 1-mm^2 area within or adjacent to the artery lumen and clearly distinguishable from the vessel lumen. If no narrowing

© 2022 Pan Y et al. *JAMA Network Open.*

was identified, the plaque was considered to have no detectable stenosis. For each territory, area-based degree of narrowing was recorded for the most stenotic plaque according to the Society of Cardiovascular Computed Tomography criteria.[7] Percent stenosis = [(1- (MLA / NLA))] ×100%, where MLA = the lumen area of the artery at the site of the most severe degree of stenosis, and NLA = the lumen area of the proximal normal artery. If the proximal artery was diseased (e.g., renal artery origin stenosis), the lumen area of the distal portion of the artery at its widest, parallel, non-tortuous normal segment was substituted (second choice). Non-atherosclerotic stenosis, such as narrowing caused by myocardial bridging and physiological stenosis, were not included in this analysis. Each plaque was also characterized as calcified or noncalcified. The interobserver agreement for the detection of plaque and stenosis was evaluated by replicating the read of a random sample of 120 participants. Good reproducibility was found for the presence of plaque and artery stenosis in all territories (Cohen κ= 0.96 and 0.92 for coronary artery, Cohen κ= 0.96 and 0.64 for subclavian artery, Cohen κ= 1.00 and 1.00 for aortas, Cohen κ= 1.00 and 0.85 for renal artery, Cohen κ= 1.00 and 0.89 for iliofemoral artery).

For coronary arteries, participants with ≥50% stenosis were further classified as having 1-, 2- or 3-vessel disease and whether they had left main or proximal left anterior descending disease.[8] The following anatomical assumptions were made to define the three main coronary vessels: Left anterior descending artery (LAD): left main, left descending artery, diagonal artery and septal branch. Left circumflex artery (CXA): left circumflex artery and obtuse margin. Right coronary artery (RCA): right coronary artery, posterior lateral branches and right posterior descending artery. One-vessel, two-vessel or three-vessel disease was then defined by a ≥50% stenosis in one, two or three of the above vessels.

© 2022 Pan Y et al. *JAMA Network Open.*

Coronary artery evaluation:

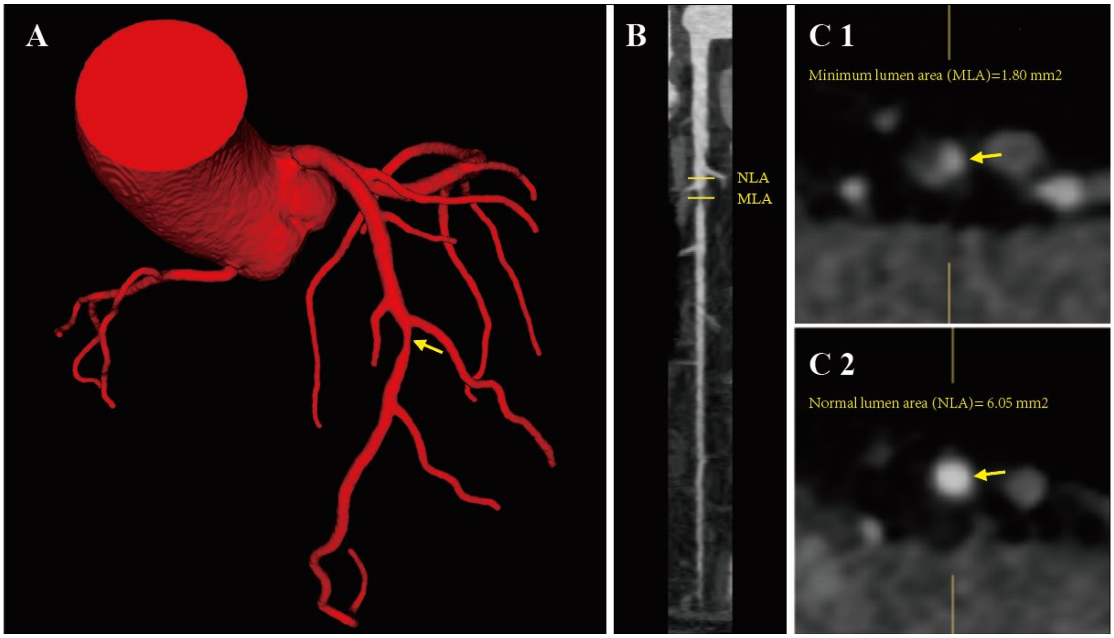

CTA showed stenosis in left anterior descending coronary artery. Percent stenosis = [(1-(1.80mm^2 / 6.05mm^2)] ×100% = 70.2%.

Subclavian artery evaluation:

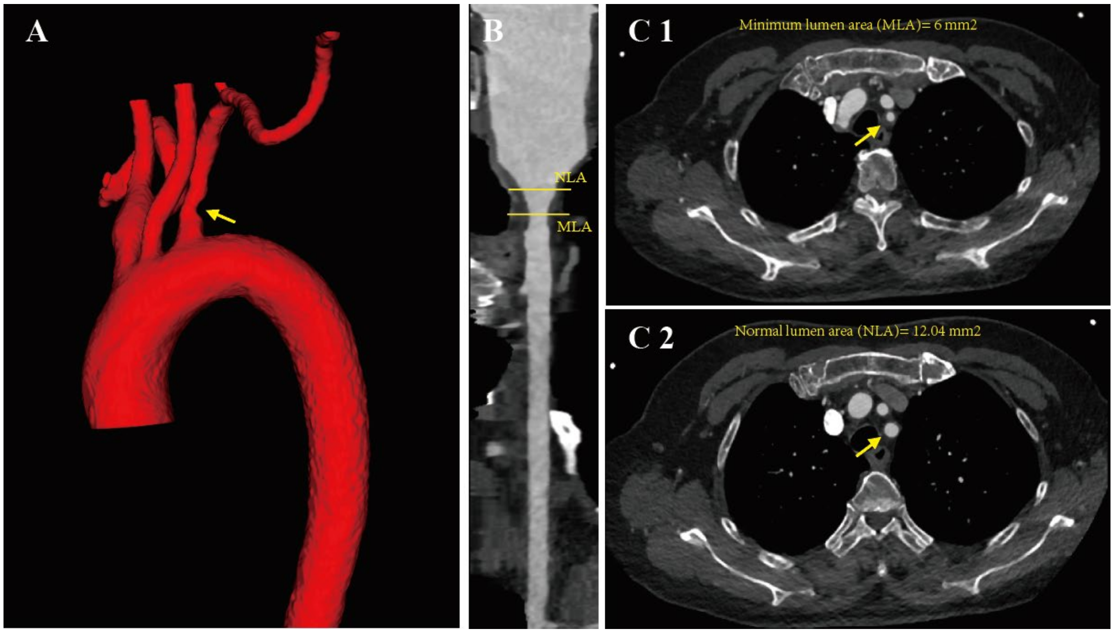

CTA showed stenosis in left subclavian artery. Percent stenosis = [(1- (6 mm^2 / 12.04 mm^2)] ×100% = 50.2%.

© 2022 Pan Y et al. *JAMA Network Open.*

Aorta evaluation:

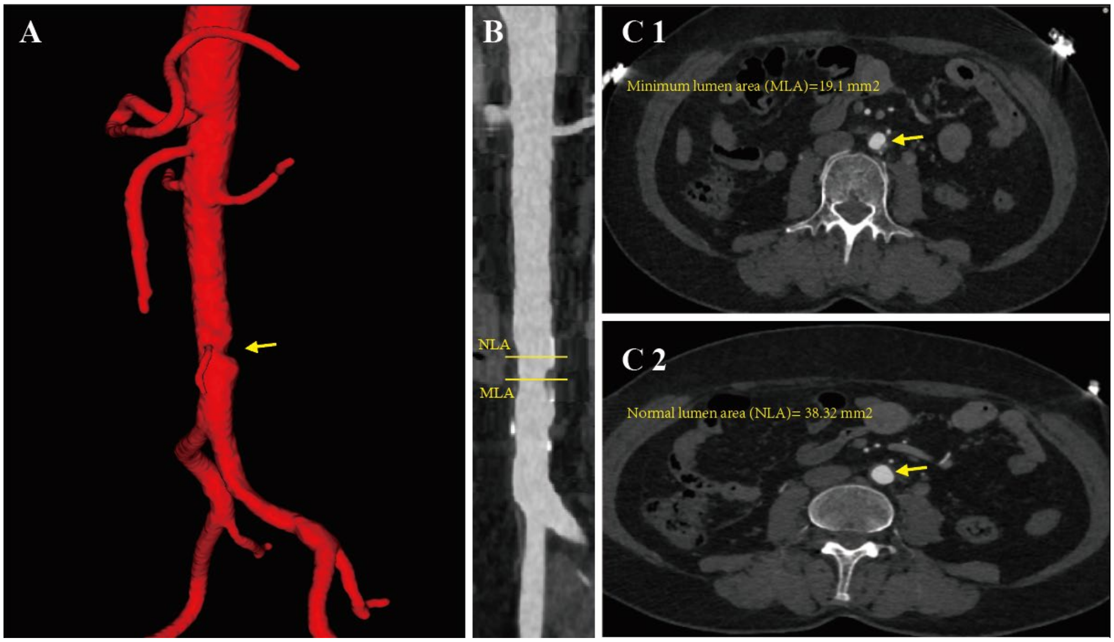

CTA showed stenosis in abdominal aorta. Percent stenosis = [(1- (19.1 mm^2 / 38.32 mm^2)) ×100% = 50.2%.

Renal artery evaluation:

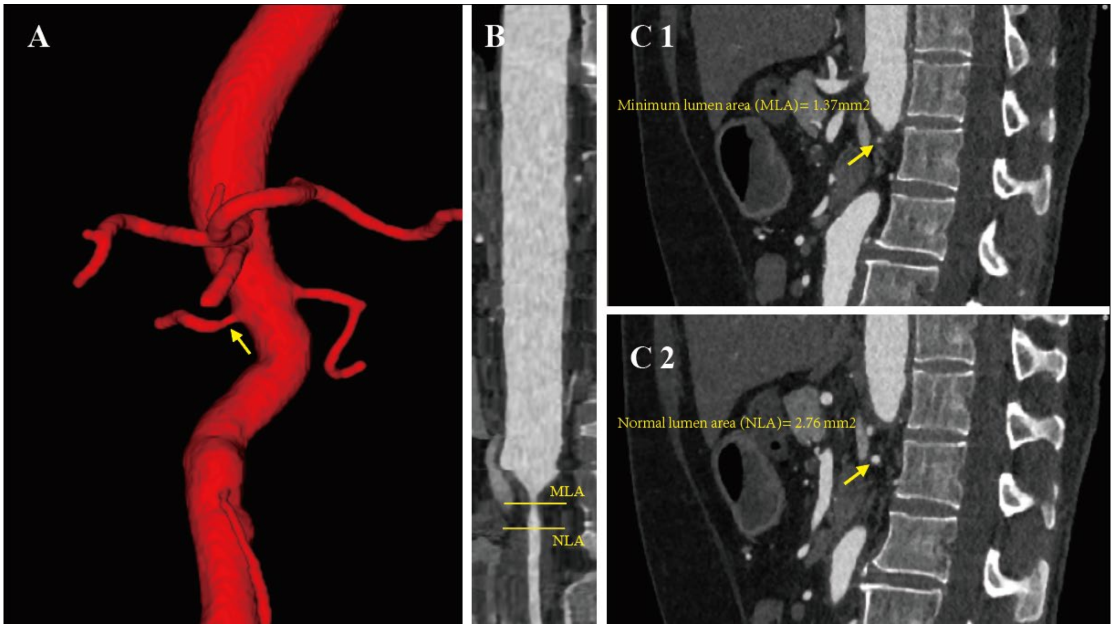

CTA showed stenosis in right renal artery. Percent stenosis = [(1- (1.37 mm^2 /2.76 mm^2)) ×100% = 50.4%.

© 2022 Pan Y et al. *JAMA Network Open.*

Iliofemoral artery evaluation:

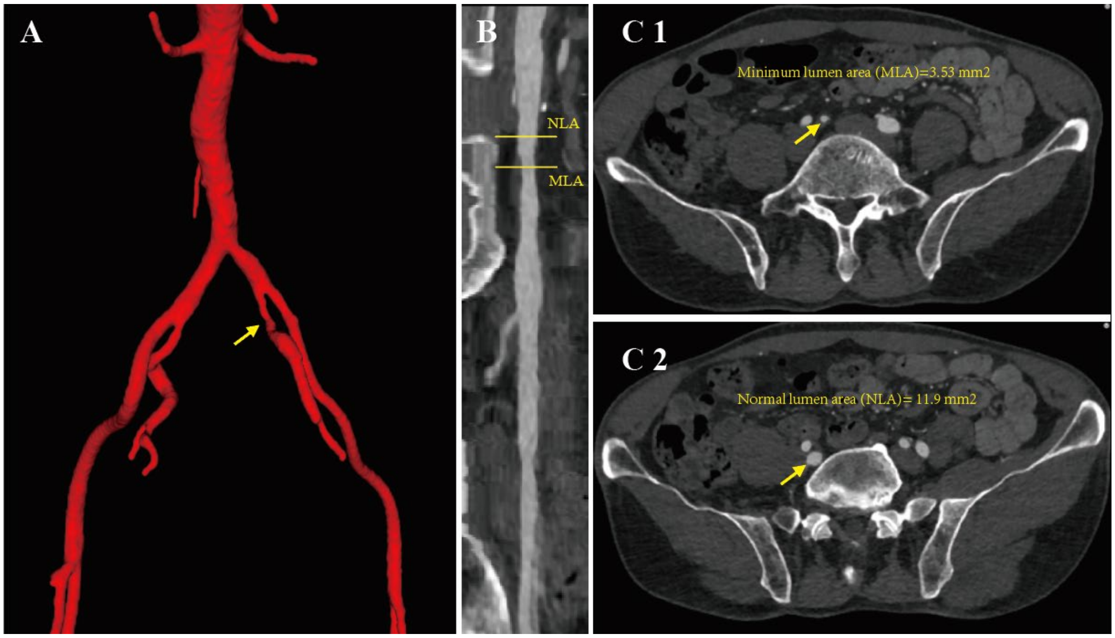

CTA showed stenosis in left iliac artery. Percent stenosis = $[(1- (3.53\ mm^2 / 11.9\ mm^2)] \times 100\%$ = 70.3%.

© 2022 Pan Y et al. *JAMA Network Open.*

eFigure 1. Prevalence and Vascular Distribution of Plaque and Arterial Stenosis in Individuals Without History of ASCVD

Abbreviations: ASCVD, atherosclerotic cardiovascular disease.

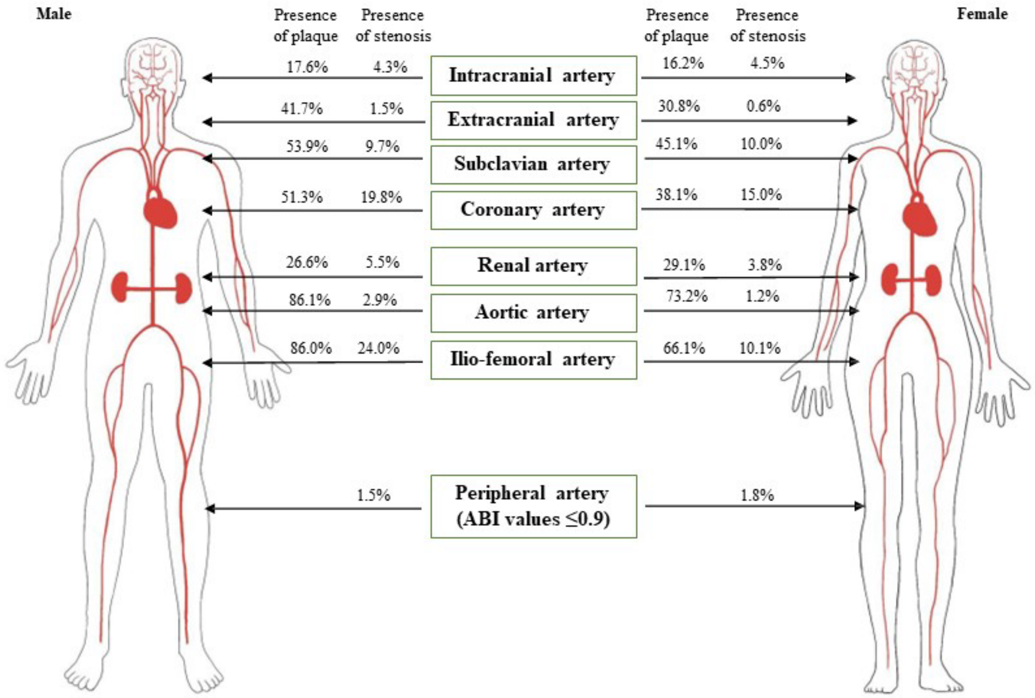

© 2022 Pan Y et al. *JAMA Network Open.*

eFigure 2. Burden of Multiterritorial Plaque and Arterial Stenosis by Age and Sex in Individuals Without History of ASCVD

Abbreviations: ASCVD, atherosclerotic cardiovascular disease.

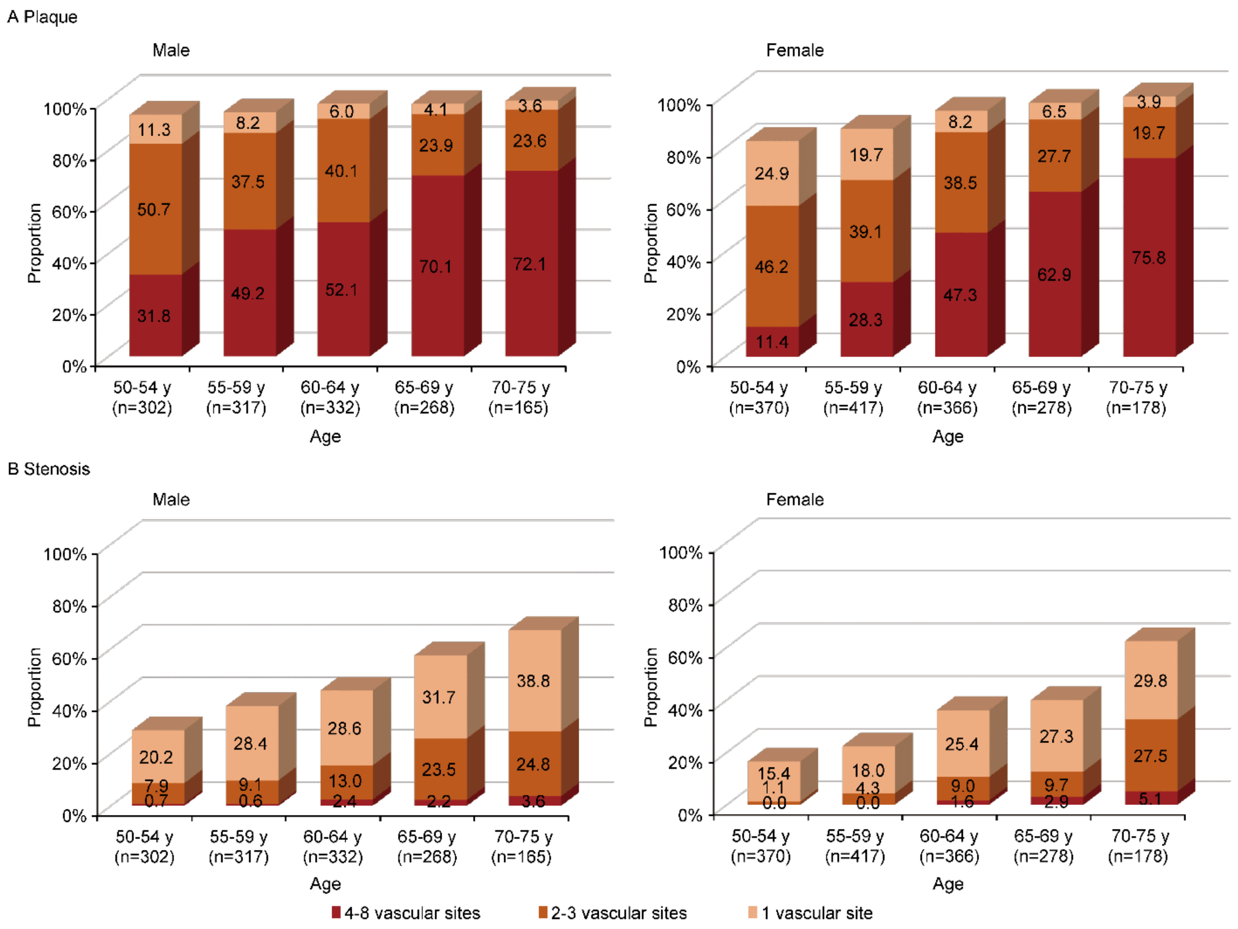

© 2022 Pan Y et al. *JAMA Network Open.*

eTable 1. Comparison of Participant Characteristics of Responders and Nonresponders for the PRECISE Study

Characteristics	Responders, No(%) (N=3067)	Non-responders, No(%) (N=1135)	p value
Sex Male	1427(46.5)	536(47.2)	0.69
Female	1640(53.5)	599(52.8)	
Age, mean (SD), y	61.2±6.7	61.1±6.5	0.62

PRECISE, PolyvasculaR Evaluation for Cognitive Impairment and vaScular Events; SD, standard deviation.

© 2022 Pan Y et al. *JAMA Network Open.*

eTable 2. Comparison of Participant Characteristics in the PRECISE Study and Nationwide Data

Characteristics	PRECISE	Nationwide	Sources of nationwide data
Female	53.5%	50.0%	China Statistical Yearbook 2018:[9] national sampling survey data for subjects of 50-74 years in 2017.
Age, y			
50-54	22.1%	31.3%	
55-59	24.3%	19.4%	
60-64	23.0%	22.0%	
65-69	18.7%	16.7%	
70-74	11.9%	10.6%	
Medical history			
Stroke	2.8%	3.5%	Wang et al. Nationwide population-based stroke survey in 2013 (N=179,527; 50-79 yrs)[10]
Diabetes mellitus	21.6%	20.2%	Wang et al. Nationwide population-based diabetes survey in 2013 (N=49,542; ≥60 yrs)[11]
Hypertension	43.1%	42.0%	Wang et al. China Hypertension Survey in 2012-2015 (N=197,609; 45-74 yrs)[12]

Abbreviations: PRECISE, PolyvasculaR Evaluation for Cognitive Impairment and vaScular Events.

© 2022 Pan Y et al. *JAMA Network Open.*

eTable 3. Prevalence of Cardiovascular Risk Factors and ASCVD, Stratified by Age and Sex

	Men, No(%)						Women, No(%)				
	**50-54 y (n=304)**	**55-59 y (n=325)**	**60-64 y (n=337)**	**65-69 y (n=285)**	**70-75 y (n=176)**		**50-54 y (n=375)**	**55-59 y (n=419)**	**60-64 y (n=369)**	**65-69 y (n=287)**	**70-75 y (n=190)**
Vascular risk factors											
Smoking	218(71.7)	247(76.0)	243(72.1)	194(68.1)	122(69.3)		0(0.0)	0(0.0)	1(0.3)	0(0.0)	1(0.5)
Former smoker	56(18.4)	83(25.5)	93(27.6)	89(31.2)	75(42.6)		0(0.0)	0(0.0)	1(0.3)	0(0.0)	0(0.0)
Current smoker	162(53.3)	164(50.5)	150(44.5)	105(36.8)	47(26.7)		0(0.0)	0(0.0)	0(0.0)	0(0.0)	1(0.5)
Family history of ASCVD	65(21.4)	74(22.8)	68(20.2)	48(16.8)	25(14.2)		80(21.3)	96(22.9)	79(21.4)	53(18.5)	28(14.7)
Hypertension	92(30.3)	114(35.1)	140(41.5)	147(51.6)	103(58.5)		110(29.3)	152(36.3)	180(48.8)	155(54.0)	128(67.4)
Diabetes mellitus	53(17.4)	58(17.8)	68(20.2)	59(20.7)	58(33.0)		52(13.9)	67(16.0)	90(24.4)	92(32.1)	66(34.7)
Dyslipidemia	151(49.7)	136(41.8)	127(37.7)	110(38.6)	74(42.0)		122(32.5)	161(38.4)	174(47.2)	133(46.3)	94(49.5)
No. of risk factors*											
0	20(6.6)	26(8.0)	26(7.7)	21(7.4)	5(2.8)		149(39.7)	113(27.0)	75(20.3)	56(19.5)	30(15.8)
1	95(31.3)	84(25.8)	101(30.0)	84(29.5)	45(25.6)		126(33.6)	179(42.7)	136(36.9)	91(31.7)	59(31.1)
2	107(35.2)	131(40.3)	115(34.1)	95(33.3)	59(33.5)		64(17.1)	86(20.5)	96(26.0)	85(29.6)	54(28.4)
≥3	82(27.0)	84(25.8)	95(28.2)	85(29.8)	67(38.1)		36(9.6)	41(9.8)	62(16.8)	55(19.2)	47(24.7)
ASCVD**	2(0.7)	8(2.5)	5(1.5)	17(6.0)	11(6.3)		5(1.3)	2(0.5)	3(0.8)	9(3.1)	12(6.3)

Abbreviations: ASCVD, atherosclerotic cardiovascular disease.
* Risk factors included smoking (former or current smoker), family history of ASCVD, hypertension, diabetes mellitus and dyslipidemia.
** ASCVD included history of ischemic stroke and myocardial infarction.

© 2022 Pan Y et al. *JAMA Network Open.*

eTable 4. Distribution and Characteristics of Plaque and Stenosis in Thoracoabdominal Arteries

	Participants, No (%)		
**Characteristics**	**Total**	**Men**	**Women**
Coronary arteries			
Available participants	3045	1414	1631
Any plaque	1366(44.9)	734(51.9)	632(38.7)
Any stenosis ≥50%	542(17.8)	292(20.7)	250(15.3)
One-vessel disease	391(12.8)	206(14.6)	185(11.3)
Two-vessel disease	102(3.3)	56(4.0)	46(2.8)
Three-vessel disease	49(1.6)	30(2.1)	19(1.2)
Left main disease	20(0.7)	13(0.9)	7(0.4)
Proximal LAD disease	176(5.8)	84(5.9)	92(5.6)
Left main, proximal LAD, or 3-vessel disease	203(6.7)	101(7.1)	102(6.3)
Only noncalcified plaques*	453(14.9)	222(15.7)	231(14.2)
Any plaque noncalcified**	752(24.7)	380(26.9)	372(22.8)
Any noncalcified stenosis ≥50%	270(8.9)	133(9.4)	137(8.4)
Subclavian arteries			
Available participants	3011	1396	1615
Any plaque	1500(49.8)	765(54.8)	735(45.5)
Any stenosis ≥50%	308(10.2)	140(10.0)	168(10.4)
Only noncalcified plaques*	588(19.5)	289(20.7)	299(18.5)
Any plaque noncalcified**	787(26.1)	405(29.0)	382(23.7)
Any noncalcified stenosis ≥50%	139(4.6)	70(5.0)	69(4.3)
Aorta arteries			
Available participants	3038	1412	1626
Any plaque	2419(79.6)	1221(86.5)	1198(73.7)
Any stenosis ≥50%	65(2.1)	44(3.1)	21(1.3)
Only noncalcified plaques*	557(18.3)	267(18.9)	290(17.8)
Any plaque noncalcified**	796(26.2)	386(27.3)	410(25.2)

© 2022 Pan Y et al. *JAMA Network Open.*

Any noncalcified stenosis ≥50%	3(0.1)	1(0.1)	2(0.1)
Renal arteries			
Available participants	3045	1414	1631
Any plaque	873(28.7)	388(27.4)	485(29.7)
Any stenosis ≥50%	145(4.8)	83(5.9)	62(3.8)
Only noncalcified plaques*	399(13.1)	195(13.8)	204(12.5)
Any plaque noncalcified**	509(16.7)	246(17.4)	263(16.1)
Any noncalcified stenosis ≥50%	76(2.5)	47(3.3)	29(1.8)
Iliofemoral arteries			
Available participants	3050	1416	1634
Any plaque	2312(75.8)	1223(86.4)	1089(66.6)
Any stenosis ≥50%	527(17.3)	353(24.9)	174(10.6)
Only noncalcified plaques*	200(6.6)	118(8.3)	82(5.0)
Any plaque noncalcified**	1411(46.3)	778(54.9)	633(38.7)
Any noncalcified stenosis ≥50%	195(6.4)	142(10.0)	53(3.2)

Abbreviations: LAD, left anterior descending artery. Values are present as n (%).

* Only noncalcified plaques were defined as participants who had exclusively noncalcified plaques in all affected segments.

** Any plaque noncalcified were defined as participants who had either only noncalcified plaques or a mix of noncalcified and calcified plaques.

© 2022 Pan Y et al. *JAMA Network Open.*

eTable 5. Associations of Age, Sex, Cardiovascular Risk Factors With Presence of Plaque in Individual Vascular Territories

	Intracranial artery (n=3065)	Extracranial artery (n=3049)	Coronary artery (n=3045)	Subclavian artery (n=3011)	Aortic artery (n=3038)	Renal artery (n=3045)	Ilio-femoral artery (n=3050)
Age							
50-59 y	1	1	1	1	1	1	1
60-64 y	1.46(1.14-1.88)	1.08(0.90-1.31)	2.04(1.70-2.46)	2.25(1.87-2.71)	1.95(1.56-2.44)	2.75(2.21-3.42)	2.45(1.97-3.05)
≥65 y	2.43(1.96-3.02)	1.47(1.24-1.75)	3.53(2.97-4.20)	4.00(3.35-4.78)	5.76(4.37-7.59)	5.76(4.73-7.02)	5.04(3.96-6.40)
Male sex	1.09(0.91-1.32)	1.62(1.39-1.88)	1.71(1.48-1.97)	1.45(1.26-1.68)	2.28(1.89-2.76)	0.89(0.76-1.05)	3.17(2.64-3.81)
Obesity	1.43(1.05-1.95)	1.10(0.84-1.43)	1.53(1.17-1.99)	1.33(1.02-1.74)	1.57(1.10-2.25)	1.59(1.19-2.11)	1.73(1.22-2.44)
Vascular risk factors							
Smoking	0.94(0.70-1.26)	1.12(0.89-1.42)	1.23(0.97-1.57)	1.81(1.41-2.31)	3.23(2.33-4.46)	1.36(1.03-1.81)	2.12(1.53-2.94)
Former smoker	0.97(0.68-1.38)	1.08(0.81-1.43)	1.17(0.87-1.56)	1.50(1.12-2.02)	2.48(1.64-3.76)	1.25(0.90-1.74)	1.78(1.17-2.70)
Current smoker	0.92(0.66-1.27)	1.15(0.89-1.49)	1.27(0.98-1.65)	2.03(1.55-2.66)	3.76(2.60-5.44)	1.45(1.07-1.97)	2.33(1.62-3.35)
Family history of ASCVD	1.01(0.80-1.28)	1.04(0.86-1.25)	1.18(0.98-1.43)	1.09(0.91-1.32)	1.12(0.89-1.41)	1.21(0.98-1.49)	1.13(0.90-1.41)
Hypertension	2.57(2.11-3.14)	1.18(1.01-1.38)	1.87(1.60-2.18)	2.12(1.81-2.48)	2.26(1.83-2.79)	2.74(2.31-3.26)	2.57(2.10-3.14)
Diabetes mellitus	1.80(1.46-2.22)	1.05(0.87-1.26)	1.78(1.48-2.14)	1.55(1.29-1.87)	2.36(1.78-3.12)	1.31(1.08-1.59)	2.10(1.63-2.72)
Dyslipidemia	1.42(1.17-1.71)	1.34(1.16-1.56)	1.51(1.30-1.76)	1.51(1.30-1.77)	1.83(1.50-2.23)	1.83(1.54-2.17)	2.03(1.68-2.46)
No. of risk factors*							
0	1	1	1	1	1	1	1
1	1.31(0.93-1.85)	1.13(0.89-1.42)	1.58(1.24-2.02)	1.59(1.25-2.02)	1.79(1.40-2.27)	2.39(1.74-3.30)	1.77(1.39-2.24)
2	2.10(1.49-2.95)	1.13(0.89-1.45)	2.08(1.62-2.67)	2.49(1.95-3.19)	3.71(2.79-4.94)	4.07(2.95-5.62)	3.03(2.32-3.97)
≥3	3.13(2.21-4.44)	1.63(1.26-2.11)	3.65(2.79-4.78)	3.57(2.73-4.67)	5.53(3.88-7.89)	5.41(3.87-7.56)	6.31(4.45-8.97)

Abbreviations: ASCVD, atherosclerotic cardiovascular disease; SD, standard deviation. Data are expressed as odds ratios with their confidence intervals adjusted by age and sex and calculated with logistic regression models.

* Risk factors included smoking (former or current smoker), family history of ASCVD, hypertension, diabetes mellitus and dyslipidemia.

© 2022 Pan Y et al. *JAMA Network Open.*

eTable 6. Associations of Age, Sex, Cardiovascular Risk Factors With Presence of Arterial Stenosis in Individual Vascular Territories

	Intracranial artery (n=3065)	Extracranial artery (n=3049)	Coronary artery (n=3045)	Subclavian artery (n=3011)	Aortic artery (n=3038)	Renal artery (n=3045)	Ilio-femoral artery (n=3050)	Ankle-brachial index ≤0.9 (n=3047)
Age								
50-59 y	1	1	1	1	1	1	1	1
60-64 y	1.49(0.90-2.47)	2.61(0.97-7.04)	1.95(1.52-2.51)	1.90(1.37-2.64)	2.61(1.32-5.16)	3.89(2.24-6.75)	1.91(1.47-2.48)	0.89(0.45-1.78)
≥65 y	3.45(2.31-5.14)	3.74(1.54-9.04)	3.15(2.52-3.93)	3.13(2.36-4.15)	3.22(1.73-6.00)	7.31(4.47-11.97)	3.70(2.95-4.64)	0.56(0.27-1.16)
Male sex	0.98(0.70-1.38)	2.67(1.27-5.63)	1.44(1.19-1.73)	0.96(0.76-1.22)	2.46(1.45-4.15)	1.58(1.13-2.21)	2.79(2.29-3.40)	0.79(0.44-1.40)
Obesity	1.06(0.59-1.91)	2.62(1.07-6.46)	1.12(0.81-1.56)	1.16(0.77-1.74)	0.93(0.37-2.35)	1.61(0.95-2.72)	1.16(0.82-1.64)	0.91(0.32-2.54)
Vascular risk factors								
Smoking	0.94(0.55-1.61)	1.97(0.67-5.80)	1.37(1.01-1.85)	1.40(0.93-2.11)	4.16(1.56-11.31)	1.72(0.99-2.98)	2.74(1.99-3.77)	2.14(0.64-7.18)
Former smoker	0.83(0.43-1.59)	1.90(0.57-6.32)	1.38(0.97-1.96)	1.40(0.87-2.24)	2.75(0.91-8.35)	1.48(0.79-2.78)	1.85(1.27-2.68)	0.34(0.04-3.30)
Current smoker	1.04(0.57-1.87)	2.04(0.65-6.43)	1.36(0.98-1.89)	1.40(0.90-2.20)	5.35(1.92-14.90)	1.93(1.07-3.49)	3.59(2.55-5.05)	3.20(0.94-10.89)
Family history of ASCVD	1.16(0.76-1.75)	1.66(0.76-3.61)	1.00(0.79-1.27)	1.08(0.80-1.46)	1.84(1.06-3.18)	1.30(0.86-1.97)	0.99(0.77-1.27)	0.86(0.42-1.79)
Hypertension	2.61(1.80-3.78)	2.71(1.26-5.81)	1.66(1.36-2.01)	1.48(1.16-1.89)	2.27(1.33-3.86)	4.13(2.73-6.24)	2.33(1.90-2.86)	1.77(0.99-3.15)
Diabetes mellitus	1.78(1.24-2.55)	4.64(2.29-9.40)	1.64(1.33-2.03)	1.03(0.78-1.37)	1.66(0.97-2.82)	1.43(0.99-2.07)	2.11(1.69-2.62)	0.78(0.36-1.69)
Dyslipidemia	1.31(0.93-1.83)	2.16(1.07-4.38)	1.43(1.18-1.73)	1.49(1.17-1.89)	1.86(1.13-3.07)	1.68(1.19-2.36)	1.86(1.52-2.28)	0.57(0.30-1.06)
No. of risk factors*								
0	1	1	1	1	1	1	1	1
1	3.00(1.33-6.78)	NE**	1.48(1.04-2.11)	1.33(0.87-2.05)	1.47(0.30-7.17)	7.30(1.74-30.72)	1.95(1.21-3.13)	0.58(0.25-1.31)
2	3.91(1.73-8.80)	NE**	1.92(1.35-2.74)	1.78(1.16-2.74)	5.12(1.19-22.14)	12.01(2.89-49.93)	3.49(2.19-5.54)	0.82(0.36-1.87)
≥3	5.05(2.22-11.48)	NE**	2.92(2.03-4.19)	2.17(1.40-3.38)	8.64(2.01-37.12)	18.62(4.48-77.32)	6.92(4.36-11.00)	0.93(0.38-2.26)

Abbreviations: ASCVD, atherosclerotic cardiovascular disease; SD, standard deviation. Data are expressed as odds ratios with their confidence intervals adjusted by age and sex and calculated with logistic regression models.

© 2022 Pan Y et al. *JAMA Network Open.*

* Risk factors included smoking (former or current smoker), family history of ASCVD, hypertension, diabetes mellitus and dyslipidemia.

** odds ratios were not estimated due to no subject having stenosis in the group of individuals without risk factor.

© 2022 Pan Y et al. *JAMA Network Open.*

eTable 7. Associations Between the Presence of Atherosclerosis in Individual Vascular Territories and ASCVD

	ASCVD* in those with vs. without atherosclerosis in the artery, No/N (%)	OR (95% CI)	P value
Intracranial artery	37/542(6.8) vs. 37/2523(1.5)	3.91(2.43-6.28)	<0.001
Extracranial artery	42/1110(3.8) vs. 30/1939(1.5)	2.15(1.32-3.48)	0.002
Coronary artery	52/1366(3.8) vs. 22/1679(1.3)	1.99(1.18-3.35)	0.01
Subclavian artery	55/1500(3.7) vs. 18/1511(1.2)	2.04(1.17-3.57)	0.01
Aortic artery	73/2419(3.0) vs. 1/619(0.2)	11.31(1.55-82.33)	0.02
Renal artery	42/873(4.8) vs. 32/2172(1.5)	2.21(1.34-3.62)	0.002
Ilio-femoral artery	71/2312(3.1) vs. 3/738(0.4)	4.44(1.37-14.43)	0.01
Ankle-brachial index ≤0.9	0/49(0.0) vs. 73/2998(2.4)	NE	NE

Abbreviations: ASCVD, atherosclerotic cardiovascular disease; CI, confidence interval; OR, odds ratio; NE, not estimable.

ORs were calculated using logistic regression models adjusted for age and sex.

* ASCVD included history of ischemic stroke and myocardial infarction.

© 2022 Pan Y et al. *JAMA Network Open.*

eTable 8. Demographic Characteristics and Risk Factors by Number of Vascular Territories With Plaque

	Participants, No (%)				
Characteristics	None (n=197)	Single Territory (n=329)	2-3 Territories (n=1105)	4-8 Territories (n=1436)	P value
Age, mean (SD), y	56.7±4.8	57.7±5.6	59.6±6.2	63.8±6.4	<0.001
Male sex	47(23.9)	98(29.8)	514(46.5)	768(53.5)	<0.001
BMI, mean (SD), kg/m^2	22.7±2.8	23.2±3.0	23.7±3.0	24.1±3.0	<0.001
SBP, mean (SD), mm Hg	120.6±13.6	121.5±15.1	126.9±15.2	134.1±16.2	<0.001
DBP, mean (SD), mm Hg	72.7±8.8	72.6±9.5	75.0±9.0	76.3±8.9	<0.001
Vascular risk factors					
Smoking	26(13.2)	56(17.0)	354(32.0)	590(41.1)	<0.001
Former smoker	11(5.6)	20(6.1)	135(12.2)	231(16.1)	<0.001
Current smoker	15(7.6)	36(10.9)	219(19.8)	359(25.0)	
Family history of ASCVD	35(17.8)	57(17.3)	237(21.4)	287(20.0)	0.40
Hypertension	43(21.8)	64(19.5)	370(33.5)	844(58.8)	<0.001
Diabetes mellitus	16(8.1)	32(9.7)	215(19.5)	400(27.9)	<0.001
Dyslipidemia	48(24.4)	98(29.8)	419(37.9)	717(49.9)	<0.001
TC ≥6.24 mmol/L	19(9.6)	33(10.0)	160(14.5)	294(20.5)	<0.001
LDL-C ≥4.16 mmol/L	4(2.0)	6(1.8)	38(3.4)	89(6.2)	<0.001
HDL-C <1.04 mmol/L	16(8.1)	36(10.9)	158(14.3)	259(18.0)	<0.001
No. of risk factors*					<0.001
0	81(41.1)	112(34.0)	219(19.8)	109(7.6)	
1	75(38.1)	143(43.5)	394(35.7)	388(27.0)	
2	30(15.2)	61(18.5)	319(28.9)	482(33.6)	
≥3	11(5.6)	13(4.0)	173(15.7)	457(31.8)	
Treatment					
Antihypertensive therapy	28(14.2)	32(9.7)	195(17.6)	568(39.6)	<0.001
Antidiabetic therapy	3(1.5)	11(3.3)	75(6.8)	185(12.9)	<0.001
Lipid-lowering therapy	2(1.0)	5(1.5)	22(2.0)	91(6.3)	<0.001
ASCVD**	0(0.0)	3(0.9)	10(0.9)	61(4.2)	<0.001

Abbreviations: BMI, body mass index; SBP, Systolic blood pressure; DBP, Diastolic blood pressure; TC, Total Cholesterol; HDL-C, High Density Lipoprotein Cholesterol; LDL-C, Low Density Lipoprotein Cholesterol; ASCVD, atherosclerotic cardiovascular disease.

© 2022 Pan Y et al. *JAMA Network Open.*

* Risk factors included smoking (former or current smoker), family history of ASCVD, hypertension, diabetes mellitus and dyslipidemia.

** ASCVD included history of ischemic stroke and myocardial infarction.

© 2022 Pan Y et al. *JAMA Network Open.*

eTable 9. Demographic Characteristics and Risk Factors by Number of Vascular Territories With Arterial Stenosis

	Participants, No (%)				
**Characteristics**	**None (n=1887)**	**Single Territory (n=768)**	**2-3 Territories (n=357)**	**4-8 Territories (n=55)**	**P value**
Age, mean (SD), y	59.7±6.1	62.6±6.7	65.3±6.6	67.4±5.5	<0.001
Male sex	776(41.1)	405(52.7)	216(60.5)	30(54.5)	<0.001
BMI, mean (SD), kg/m^2	23.7±3.0	23.8±3.1	24.0±3.0	24.2±3.5	0.23
SBP, mean (SD), mm Hg	126.7±15.6	131.5±15.9	136.4±16.7	142.9±18.6	<0.001
DBP, mean (SD), mm Hg	74.6±9.0	75.9±9.0	76.4±8.9	77.7±9.3	<0.001
Vascular risk factors					
Smoking	530(28.1)	287(37.4)	181(50.7)	28(50.9)	<0.001
Former smoker	212(11.2)	102(13.3)	73(20.4)	10(18.2)	<0.001
Current smoker	318(16.9)	185(24.1)	108(30.3)	18(32.7)	
Family history of ASCVD	371(19.7)	174(22.7)	57(16.0)	14(25.5)	0.04
Hypertension	667(35.3)	362(47.1)	246(68.9)	46(83.6)	<0.001
Diabetes mellitus	319(16.9)	206(26.8)	113(31.7)	25(45.5)	<0.001
Dyslipidemia	707(37.5)	348(45.3)	195(54.6)	32(58.2)	<0.001
TC ≥6.24 mmol/L	277(14.7)	144(18.8)	72(20.2)	13(23.6)	0.005
LDL-C ≥4.16 mmol/L	76(4.0)	40(5.2)	16(4.5)	5(9.1)	0.20
HDL-C <1.04 mmol/L	242(12.8)	134(17.4)	76(21.3)	17(30.9)	<0.001
No. of risk factors*					<0.001
0	415(22.0)	90(11.7)	16(4.5)	0(0.0)	
1	680(36.0)	239(31.1)	75(21.0)	6(10.9)	
2	524(27.8)	224(29.2)	126(35.3)	18(32.7)	
≥3	268(14.2)	215(28.0)	140(39.2)	31(56.4)	
Treatment					
Antihypertensive therapy	384(20.3)	235(30.6)	169(47.3)	35(63.6)	<0.001
Antidiabetic therapy	110(5.8)	83(10.8)	66(18.5)	15(27.3)	<0.001
Lipid-lowering therapy	61(3.2)	28(3.6)	27(7.6)	4(7.3)	<0.001
ASCVD**	21(1.1)	19(2.5)	26(7.3)	8(14.5)	<0.001

© 2022 Pan Y et al. *JAMA Network Open.*

Abbreviations: BMI, body mass index; SBP, Systolic blood pressure; DBP, Diastolic blood pressure; TC, Total Cholesterol; HDL-C, High Density Lipoprotein Cholesterol; LDL-C, Low Density Lipoprotein Cholesterol; ASCVD, atherosclerotic cardiovascular disease.

* Risk factors included smoking (former or current smoker), family history of ASCVD, hypertension, diabetes mellitus and dyslipidemia.

** ASCVD included history of ischemic stroke and myocardial infarction.

© 2022 Pan Y et al. *JAMA Network Open.*

eReferences.

1. Qiao Y, Guallar E, Suri FK, et al. MR Imaging Measures of Intracranial Atherosclerosis in a Population-based Study. *Radiology*. 2016;280(3):860-868. doi: 10.1148/radiol.2016151124
2. Samuels OB, Joseph GJ, Lynn MJ, Smith HA, Chimowitz MI. A standardized method for measuring intracranial arterial stenosis. *AJNR Am J Neuroradiol*. 2000;21(4):643-646.
3. Fox AJ. How to measure carotid stenosis. *Radiology*. 1993;186(2):316-318. doi: 10.1148/radiology.186.2.8421726
4. Kong B, Wang X, Bai J, et al. Learning tree-structured representation for 3D coronary artery segmentation. *Comput Med Imaging Graph*. 2020;80:101688. doi: 10.1016/j.compmedimag.2019.101688
5. Guo Z, Bai J, Lu Y, et al. DeepCenterline: A Multi-task Fully Convolutional Network for Centerline Extraction. In: Information Processing in Medical Imaging. 2019. arXiv:1903.10481. doi:
6. Arabi H, Zaidi H. Deep learning-based metal artefact reduction in PET/CT imaging. *Eur Radiol*. 2021;31(8):6384-6396. doi: 10.1007/s00330-021-07709-z
7. Wu FZ, Wu MT. 2014 SCCT guidelines for the interpretation and reporting of coronary CT angiography: a report of the Society of Cardiovascular Computed Tomography Guidelines Committee. *J Cardiovasc Comput Tomogr*. 2015;9(2):e3. doi: 10.1016/j.jcct.2015.01.003
8. Bergström G, Persson M, Adiels M, et al. Prevalence of Subclinical Coronary Artery Atherosclerosis in the General Population. *Circulation*. 2021;144(12):916-929. doi: 10.1161/CIRCULATIONAHA.121.055340
9. National Bureau of Statistics of China. China Statistical Yearbook 2018. China Statistics Press. Beijing (2018) (in Chinese).
10. Wang W, Jiang B, Sun H, et al. Prevalence, Incidence and Mortality of Stroke in China: Results from a Nationwide Population-Based Survey of 480,687 Adults. *Circulation*. 2017;135(8):759-771. doi: 10.1161/CIRCULATIONAHA.116.025250
11. Wang L, Gao P, Zhang M, et al. Prevalence and Ethnic Pattern of Diabetes and Prediabetes in China in 2013. *JAMA*. 2017;317(24):2515-2523. doi: 10.1001/jama.2017.7596
12. Wang Z, Chen Z, Zhang L, et al. Status of Hypertension in China: Results From the China Hypertension Survey, 2012-2015. *Circulation*. 2018;137(22):2344-2356. doi: 10.1161/CIRCULATIONAHA.117.032380

© 2022 Pan Y et al. *JAMA Network Open.*
